# Mitochondrial Transport in Glycolysis and Gluconeogenesis: Achievements and Perspectives

**DOI:** 10.3390/ijms222312620

**Published:** 2021-11-23

**Authors:** Salvatore Passarella, Avital Schurr, Piero Portincasa

**Affiliations:** 1 Department of Biomedical Sciences and Human Oncology, University of Bari “Aldo Moro”, 70124 Bari, Italy; 2 Department of Anesthesiology and Perioperative Medicine, School of Medicine, University of Louisville, Louisville, KY 40202, USA; avital.schurr@gmail.com; 3 Clinica Medica “A. Murri”, Department of Biomedical Sciences and Human Oncology, University of Bari “Aldo Moro”, 70124 Bari, Italy; piero.portincasa@uniba.it

**Keywords:** mitochondrial transport, glycolysis, gluconeogenesis, mitochondrial shuttles, phosphoenolpyruvate, L-lactate

## Abstract

Some metabolic pathways involve two different cell components, for instance, cytosol and mitochondria, with metabolites traffic occurring from cytosol to mitochondria and vice versa, as seen in both glycolysis and gluconeogenesis. However, the knowledge on the role of mitochondrial transport within these two glucose metabolic pathways remains poorly understood, due to controversial information available in published literature. In what follows, we discuss achievements, knowledge gaps, and perspectives on the role of mitochondrial transport in glycolysis and gluconeogenesis. We firstly describe the experimental approaches for quick and easy investigation of mitochondrial transport, with respect to cell metabolic diversity. In addition, we depict the mitochondrial shuttles by which NADH formed in glycolysis is oxidized, the mitochondrial transport of phosphoenolpyruvate in the light of the occurrence of the mitochondrial pyruvate kinase, and the mitochondrial transport and metabolism of L-lactate due to the L-lactate translocators and to the mitochondrial L-lactate dehydrogenase located in the inner mitochondrial compartment.

## 1. Introduction

Mitochondria play a key role in cell metabolism, and they govern a significant cross talk with the cytosol. This task is achieved by sharing metabolic pathways which rely on both cytosolic and mitochondrial enzymes. Mitochondria export metabolites/ATP for cytosol anaplerosis and import metabolite/ADP for final oxidation and ATP synthesis. Given that mitochondria are “close spaces” within the cytosol, they possess several translocators involved in cytosol communication with the inner mitochondrial compartments. However, as reported by Taylor [1] “the transport selectivities of many carriers remain unknown, and most have not been functionally investigated in mammalian cells”. A review described “the multifaced contributions of mitochondria to cellular metabolism” but the transport and metabolism of certain metabolites in mitochondria, including L- and D-lactate and glutamine, were ignored or incompletely reported [2]. Papers describing the role of mitochondria in cancer do not take into consideration key transport processes, e.g., L-lactate which is the main product of cancer cell energy metabolism [3,4,5,6,7,8].

In what follows we describe the metabolite trafficking occurring across the mitochondrial inner membrane. We focus on aspects of energy metabolism, glycolysis, and gluconeogenesis and provide answers to questions not yet addressed or without a definite conclusion. This review is dedicated to biochemists working on novel transport processes reported in the last two decades, as well as other pathways not included in previous reviews [9,10,11,12]. Certain issues are revisited considering new experimental findings pointing to new models/hypotheses. An important point is that each cell has its own metabolism with a specific role for mitochondria. Thus, it is impossible to consider transport features of each substrate as common for mitochondria isolated from different sources. In this respect, yeast mitochondria cannot represent the ultimate model of transport and metabolism of both mammalian and plant mitochondria. Using simple and rapid methods provides better insights into the role of mitochondrial transport in the energy metabolism of a specific cell type. Both isolated, coupled mammalian mitochondria and cell/cell homogenates containing coupled mitochondria should be considered as ideal models for metabolite traffic studies. In this review we will address the following questions:

How mitochondrial traffic in energy metabolism can be quickly and easily investigated in vitro with intact, coupled mitochondria/cell homogenates containing intact, coupled mitochondria?

How cytosolic reducing equivalents, formed as NADH in glycolysis, are oxidized by mitochondria? 

Anything new about PEP transport in mitochondria in the light of the existence of pyruvate kinase (PK) in the mitochondrial matrix? 

Anything new about L-lactate mitochondrial transport and metabolism in neuronal and cancer mitochondria? Has this L-lactate-mitochondria affair reached a final conclusion? 

## 2. How Mitochondrial Traffic in Energy Metabolism Is Easily and Quickly Investigated?

The investigation of the role of mitochondrial transport in energy metabolism relies on the following aspects:

- The nature of the involved metabolites.
- The concentration of metabolites in experiments (likely using physiological concentrations).
- If and how the nature of the transport and concentration of metabolites change over time due to variations in enzymatic reactions.
- Which factors play a role in determining the specificity of a given energy metabolic pathway.

Importantly, mitochondria should be coupled and intact.

We essentially, advocate one main type of experimental approach to investigate the carrier-mediated transport in isolated mitochondria and the intramitochondrial metabolism, i.e., a “dynamic” approach investigating the kinetics of both transport and metabolism. Importantly, the closer the conditions of the experiments are to the physiological ones, the more acceptable the conclusions drawn from the experiments will be. A metabolic flux depends on its rate-limiting step, and this step must be preferentially identified and investigated. In the dynamic approach, any parameter can be plotted linearly and correlated with the concentration as a function of time, while the rate of the reaction is measured as a tangent of the progress curve. In the dynamic approach, a variety of processes can be investigated even in the absence of complete identification of the cell components involved.

In a “static” approach, experiments are performed without monitoring the metabolic flux (see Table 1). Measurements are made afterwards following inactivation procedures of the investigated molecules. These experiments include genetic, immunological analysis, confocal microscopy, magnetic resonance spectroscopy, and all the techniques in which mitochondria are incapacitated as the cell energy power. The approach provides a detailed description of the molecules involved in transport and metabolism, but no/minimal information on how certain transports and reactions can play a role in the metabolic pathways.

This section summarizes several “older, quick” methods, aimed to encourage further studies of transport aspects that deal with metabolites and/or mitochondria not yet investigated. Borrowing from the world of music, these “older” methods could be compared to Stradivarius or Guarneri violins, i.e., preferable over the modern instruments. Thus, we prefer and recommend to researchers interested in transport and metabolism to resort first to simple and rapid techniques that allow for continuous measurements of metabolite-dependent processes with isolated intact, coupled mitochondria or cell homogenates containing intact, coupled mitochondria.

### 2.1. Spectroscopic Techniques

These techniques include mainly spectrophotometry and fluorescence spectrometry [13].

Spectroscopic methods investigate mitochondrial transport and metabolism. Photometric measurements can quickly ascertain mitochondrial integrity. Notably, mitochondria can undergo changes within 1–2 h depending on the experimental conditions (e.g., temperature and pH). These changes make the comparison difficult in the same experiment since mitochondrial integrity and coupling degrees are different. Adding NADH to the mitochondria as a test of intactness is strongly recommended, as the absorbance contribution to the photometer reading is negligible. A constant slow decrease in absorbance at 334/340 nm (the peak of the reduced nicotinamide) indicates that NADH cannot, or can only poorly be oxidized by Complex I, located in the inner mitochondrial compartment. Thus, control of mitochondrial integrity can be established in a very short period and any minor contribution to NADH decrease can be considered. Evidence of the mitochondrial coupling can be obtained by checking the capability of an uncoupler to oxidize the intramitochondrial NAD(P)H through its fluorescence monitoring at 334 and 456 nm excitation and emission wavelength respectively. Having ascertained both intactness and coupling of the investigated mitochondria, specific procedures can be then used to study the mitochondrial transport related to intramitochondrial metabolism.

#### 2.1.1. Spectrophotometry

**Figure 1:**
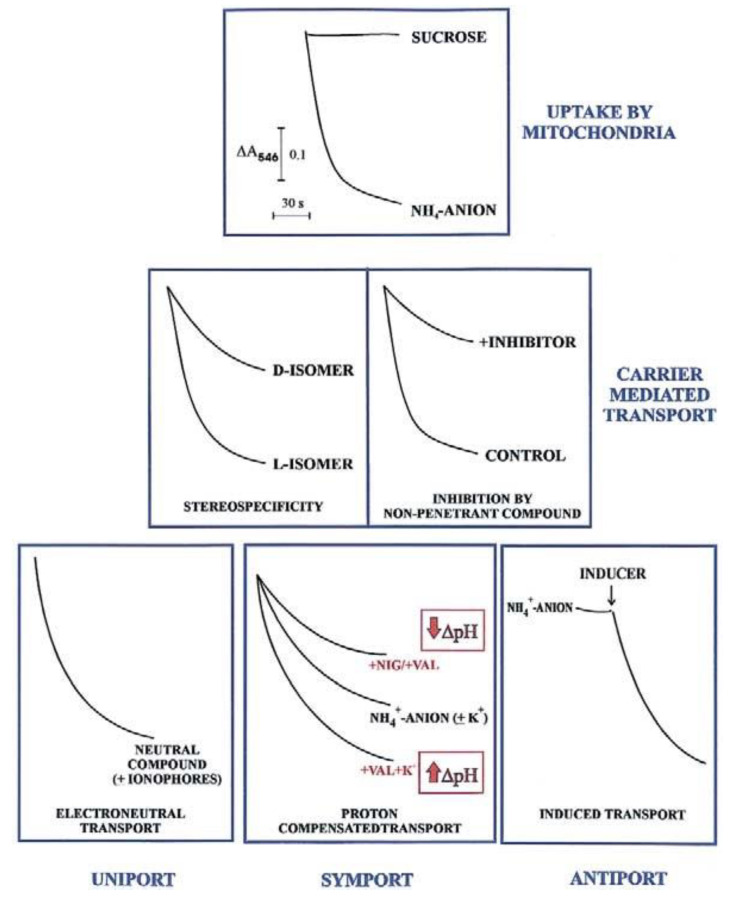
Mitochondrial swelling in transport studies, from Passarella et al. [9]. With permission from Elsevier, 2021.

In agreement with the carrier history, an initial investigation in carrier-mediated mitochondrial transport could derive by checking certain features of the swelling of mitochondria suspended in isotonic solutions of a penetrant anion (usually ammonium salt solutions). Since NH_3_ can permeate freely the mitochondrial membrane, the occurrence of mitochondrial swelling in ammonium solutions shows that the investigated anion can enter mitochondria in a proton compensated manner. Swelling is commonly monitored as a fast decrease in absorbance, usually at 546 nm, of the mitochondrial suspension that occurs osmotically with the increase in matrix solute concentration caused by mitochondrial water uptake. To show that mitochondria are intact (no swelling in sucrose isosmotic solution) and to gain a first insight into their permeability to metabolites requires less than half an hour and very small aliquots of mitochondria (about 0.1 mg protein). Following this experiment, we can answer the following question: is the investigated metabolite penetrant into mitochondria? The occurrence of swelling, however, does not indicate *per se* carrier-mediated transport, since metabolite uptake can also occur via diffusion. Nevertheless, findings such as a stereo-specific swelling, the inhibition of swelling by a non-penetrant compound, and the swelling induction by catalytic concentration of a specific metabolite, strongly indicate a carrier-mediated transport. A series of typical swelling experiments are shown in Figure 1. To ascertain whether and how the transport is energy dependent, the researcher can use certain ionophores under conditions designed to selectively affect ΔpH and ΔΨ. The presence of nigericin (NIG) or valinomycin (VAL), without added K^+^ ions, can collapse the mitochondrial ΔpH which can be increased by adding mitochondria with VAL in the presence of K^+^ ions. When swelling is not dependent on the presence of agents allowing dissipation of either ΔpH or ΔΨ (or both), the penetrant species must have no net charge, as for instance neutral amino acids.

The existence of antiport processes is suggested by the swelling occurring only as a result of the addition of a catalytic amount of one or more counter-anion/s (INDUCER) to mitochondria suspended in ammonium metabolite solutions. 

For instance, the different degrees of swelling of cerebellar granule cell mitochondria shown for L- and D-lactate (L-LAC and D-LAC) ammonium solutions [14] clearly indicate that the lactate isomer/s can enter mitochondria in different ways. Given that this cannot depend on diffusion, due to the similar structure, the existence of one or two different carrier-mediated transport can be deduced. Swelling inhibition due to a non-penetrant compound by itself indicates the occurrence of a carrier-mediated transport. Swelling studies are qualitative and of limited interest since they cannot give any indication about the rate of the transport and the amount of the metabolite taken up by mitochondria or of a substrate affinity to its carrier; nonetheless, investigation of metabolite transport using swelling measurements combined with stereo-specificity and inhibition studies is strongly recommended. This approach provides the first insight into its mitochondrial permeability in any cell.

#### 2.1.2. Spectrofluorimetry

**Figure 2:**
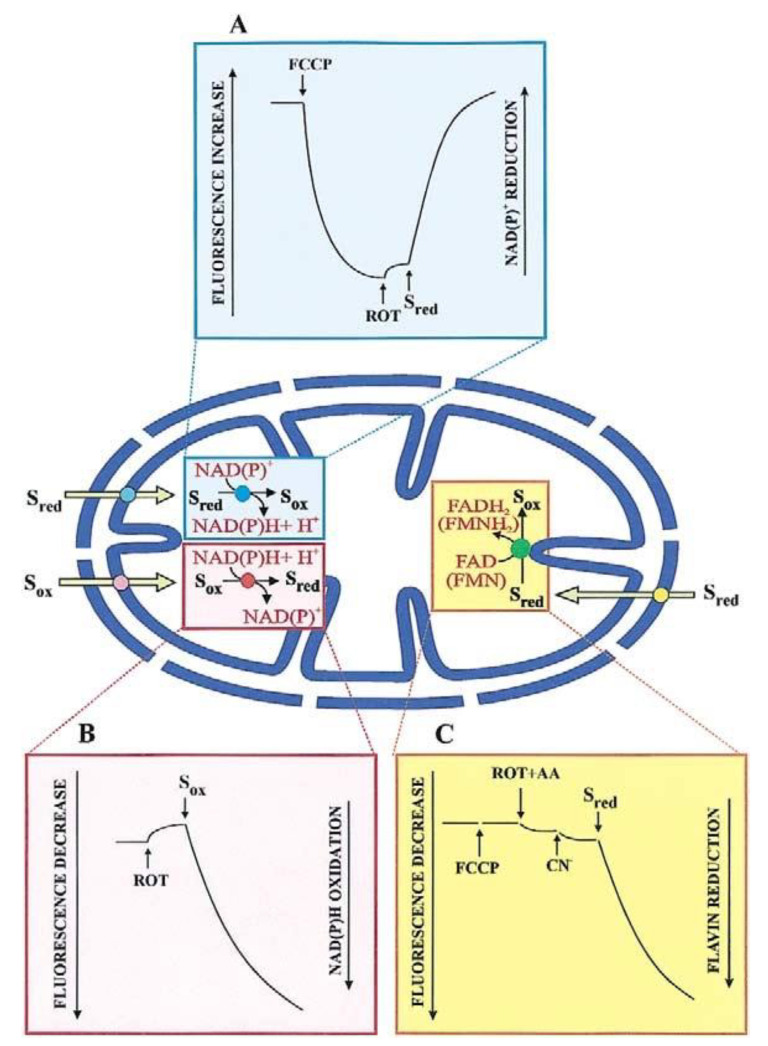
Intramitochondrial cofactor red/ox changes in transport studies. Depending on the context, fluorescence changes are studied as increase (**A**) or decrease (**B**,**C**). For details see the text. Abbreviations: AA, antimycin; CN, cyanide; FCCP, carbonyl cyanide p-trifluoromethoxy-phenylhydrazone (from Passarella et al. [9]). With permission from Elsevier, 2021.

Most substrates taken up by mitochondria, and products formed from an imported metabolite are oxidized in the mitochondrial matrix. The transport of such substrates can be monitored photometrically (with the pioneering double-beam double wavelength photometer) and fluorimetrically, via changes in the intramitochondrial cofactor red/ox state (Figure 2). Chappell and Haarhoff [15] first reported ox/red changes in the intramitochondrial pyridine nucleotides due to the uptake and metabolism of substrates catabolized by pyridine dehydrogenases. Figure 2A shows such a change where the transport of an oxidable compound is studied upon fluorescence increase, (*λ*_ex_: 334 nm; *λ*_em_: 456 nm) resulting from an intramitochondrial pyridine nucleotide reduction. 

Here, mitochondria are incubated with an uncoupler (for instance carbonyl cyanide p-trifluoro-methoxy-phenylhydrazone, FCCP), to oxidize the intramitochondrial NAD(P)H; upon attaining a constant fluorescence value rotenone is added to prevent the newly formed NAD(P)H from being oxidized by the mitochondrial complex I. An increase in fluorescence of the intramitochondrial pyridine nucleotide, if it occurs because of substrate addition outside the mitochondria, indicates that this substrate can enter the mitochondrial matrix where either its specific dehydrogenase or the dehydrogenase of a metabolite newly synthesized in the mitochondria are located. Obviously, controls are needed that no reaction of the investigated metabolite can occur outside mitochondria. An increase in fluorescence applies to malate (MAL), phosphoenolpyruvate (PEP), pyruvate (PYR), and L-lactate (L-LAC) in glycolysis. On the other hand, as first shown by Haslam and Krebs [16] the uptake of oxaloacetate (OAA), metabolized by malate dehydrogenase (MDH) in the mitochondria, is measured by the fluorescence decrease (*λ*_ex_: 334 nm; *λ*_em_: 456 nm). This is due to the intramitochondrial pyridine nucleotide oxidation, which occurs upon OAA addition to mitochondria pre-incubated with rotenone to block NADH oxidation via complex I (Figure 2B). The use of fluorimetric techniques is appropriate in the dynamic study of transport, and we agree with Mayevsky and Rogatsky [17] who state that “The large numbers of publications by different groups testify to the valuable information gathered in various experimental conditions. The monitoring of NADH levels in the tissue provides the most important information on the metabolic state of the mitochondria”. 

Surprisingly, such a simple procedure has not been appreciated and accepted in a recent paper [7] stating that “Unfortunately, some studies which have concluded the existence or absence of mitochondrial LDH, and implied the physiological relevance thereof, base such claims on experiments involving the continuous monitoring of added NADH autofluorescence in isolated mitochondria [14,18,19,20,21,22,23]”. We believe that the authors misinterpreted the cited papers since NADH was not added to mitochondria. Rather, the authors monitored only the fluorescence of the mitochondrial NAD(P)H [14,18,19,20,21,22,23].

The first demonstration of the uptake of D-LAC into mitochondria, a substrate that is metabolized by a flavin-dependent dehydrogenase, dates 2002 [24]. Mitochondria were added with FCCP and an inhibitor of the respiratory chain complex III and the fluorescence increase at wavelengths 450 and 520 nm, excitation and emission, respectively, was followed as the measurement of the substrate uptake (Figure 2C). In distinction with NAD(P)H, which is by itself fluorescent, a flavin fluorescence depends on the physiological environment due to its function as a prosthetic group.

As discussed later, the researchers must accurately ascertain that the rate of fluorescence changes mirrors the rate of the transport, rather than one of the metabolic enzymatic reactions. Thus, the application of the control strength criterion and fractional inhibition, achieved by using non-penetrant compounds, can reveal whether or not the transport is the rate-limiting step of the investigated process and can also provide an initial indication to ascertain if specific metabolite traffic depends on a single carrier or not. According to the control strength criterion, if the reciprocal of the measured rate is plotted as a function of the non-penetrant inhibitor concentration, the resulting Dixon plot (1/V against [I]), when extrapolated to zero concentration, provides a measure of transport in the absence of inhibitor; thus, the coincidence of the experimental point measured in the absence of inhibitor with the intercept indicates that the rate of inhibited step, i.e., the transport, is measured. The data from the Dixon plot could also be plotted as 1/*i* against 1/[inhibitor], where the fractional inhibition, *i*, is 1 − V_i_/V_o_ and V_i_ and V_o_ are the rates of the measured reactions in the presence or absence of inhibitor, respectively. If *y*-intercept is unity, this shows that the inhibitor can prevent the reaction completely, i.e., that the reaction cannot occur via another carrier insensitive to the inhibitor used. Obviously, control strength can be also applied to enzymatic reactions by using specific inhibitors.

A significant development of the application of spectroscopic methods to the study of mitochondrial transport was obtained at the end of the 1970s when a variety of metabolite/compound detecting systems consisting of enzyme/s and cofactor/s were developed to selectively detect the appearance outside mitochondria of molecules derived from the mitochondrial metabolism of taken-up substrates.

Interestingly, in the presence of enzymes and cofactors, the reconstruction can be made of certain metabolic pathways in which cytosol and mitochondria are involved (for refs. see [9,14]).

Since these detecting system components are mostly cytosolic, and since mitochondrial metabolism is, at least partially still occurring when they are being used, we consider this approach to be close enough to the physiological situation.

**Figure 3:**
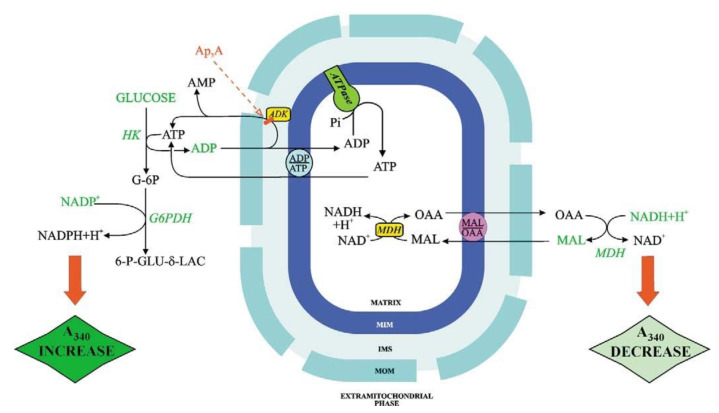
The ATP and the OAA detecting systems. The externally added components of the ATP and OAA detecting systems are reported in green. Main abbreviations: ADK, adenylate kinase; Ap_5_A, P1,P5-di(adenosine-5′)penta-phosphate; G6PDH, glucose-6-phopshate dehydrogenase; 6-P-GLU-d-LAC, 6-phosphoglucono-d-lactone; HK, hexokinase. From Passarella et al. [9]. With permission from Elsevier, 2021.

Figure 3 shows the ATP and OAA detecting systems. ATP and OAA appearance occur because of the addition of ADP and MAL, respectively, and is shown as absorbance (at 334 nm) increase and decrease, respectively. With fluorimetry (e.g., when measuring the appearance of ATP outside mitochondria, as a result of ADP addition), the transport can be studied with mitochondrial protein as low as 0.1 mg, see for instance [25].

When using a metabolite/compound detecting system the reactions occurring outside mitochondrial must not be the rate-limiting step of the whole process. Interestingly, given that ATP can be synthesized by mitochondria via both adenylate kinase and ATP synthase, the use of inhibitors that can selectively inhibit either adenylate kinase (Ap_5_A) or ATP synthase (oligomycin) can distinguish the mechanism of ATP production. Importantly, control strength application with oligomycin as an inhibitor provides a measurement of the ATP synthase reaction in intact mitochondria even if the rate-limiting step of the monitored ATP efflux is the rate of the ADP/ATP exchange. In this case, ATP synthase kinetics cannot be followed directly, however, if the rate of ATP appearance outside mitochondria due to externally added ADP is measured in the presence of oligomycin. Then the inhibition kinetics can provide information on the ATP synthesis rate: the intercept at the *Y*-axis of the linear regression of rate values obtained for oligomycin concentrations that produce inhibition of the rate of fluorescence increase represents the reciprocal of the rate of non-inhibited ADP phosphorylation via ATP synthase. By plotting the intercept values as a function of reciprocal ADP concentration, a double reciprocal plot can be obtained, which provides Vmax and Km values for ADP in the reaction catalyzed by ATP synthase [26].

Since two or more carriers can contribute to the appearance of a metabolite outside the mitochondria, specific inhibitors can be used to elucidate the mechanism by which said metabolite movement occurs. Atlante et al. [27] firstly studied the fumarate (FUM) uptake by mitochondria using protein detecting systems to monitor the appearance of a mitochondrial enzyme exported outside mitochondria. Passarella et al. [28] found that the uptake of aspartate aminotransferase into mitochondria in vitro causes efflux of MDH and *vice versa*. Other simple dynamic methods that can be used to show a metabolite mitochondrial transport and metabolism include measurements of oxygen uptake, pH changes in the extramitochondrial phase, and measurements of mitochondrial ΔΨ generation. Having established that the transport is the rate-limiting step of the monitored process, studies have shown that L-LAC uptake occurs into a variety of mitochondria, as normal prostate cells [10], cancer prostate cells [20], Hep G2 cells [21], and rabbit gastrocnemius mitochondria [22]. The different pH profiles and the different sensitivity to non-penetrant inhibitors, together with measurements of the Vmax in kinetic experiments carried out under the same experimental conditions, allow for the identification of the different carriers. For instance, evidence was found that rat liver mitochondria (RLM) possess the carriers that mediate L-LAC/H^+^ symport, L-LAC/OAA and L-LAC/PYR antiporters which proved to differ from one another and with respect to the D-LAC/H^+^ and PYR/H^+^ symporters and the D-LAC/malate and D-LAC/oxoacid antiporters [19] and that the different carriers transport glutamine in normal and acidotic rat kidney mitochondria (RKM) (see [9]).

### 2.2. Isotopic Techniques

Isotopic techniques directly study the transport, besides determining the chemical modification of a substrate into a metabolic product in a biological sample. The techniques appeared in the seventies of the last century to confirm the occurrence of metabolite transport. Direct methods for measuring metabolite transport and distribution in mitochondria have been reported in detail in the paper of Palmieri et al. [29], and here we discuss a simplified method to investigate transport kinetics, both in net uniport/symport-dependent radioactivity uptake and antiport-dependent exchanges between externally added and labelled substrates already present upon loading in mitochondria. In isotopic kinetic investigations of transport, mitochondrial metabolism must be blocked. Such blockade appears to be particularly useful since it allows for measuring only the substrate via uptake; in this case, the labeled substrate uptake is blocked using a non-penetrant inhibitor in a time range of seconds. The amount taken up is calculated by subtracting the radioactivity measured in mitochondria when the substrate is added in the presence of the inhibitor from the radioactivity measured in the organelles when the inhibitor is added a few seconds after the labeled substrate addition.

In a typical experiment, mitochondria (about 50 μL that contain 1–2 mg protein) are suspended in a standard medium in an Eppendorf cup containing tritium-labeled water (^3^H_2_O) added for measuring the pellet volume. Mitochondria (usually kept in a vial put in ice + water to prevent temperature-dependent uncoupling) are incubated for approximately 1 min to allow equilibration at the experimental chosen temperature. Next, the following are added rapidly:

A. The ^14^C-labelled substrate followed few seconds later (1–20, depending on the time when the transport is proved to be linear) by the inhibitor/s (at a concentration that should result in total inhibition) or
B. First the inhibitor/s followed by the labelled substrate.

Several seconds after these additions, the cup is placed in a microcentrifuge for 1 min to form a supernatant and a pellet (mitochondria). The supernatant is completely removed and saved, and the pellet is suspended first in 100 μL water, followed by 50 μL of 7 N perchloric acid and vigorous shaking. This treatment dissolves the mitochondria. Upon additional centrifugation, the mitochondrial content should be in the supernatant. Notice that the pellet and the obtained supernatant contain both ^14^C and ^3^H radioactivity. The ^14^C radioactivity of the taken-up substrate (n_i_) in nmol is deduced from the total radioactivity in nmol (n_tot_) minus the radioactivity in nmol attached to the mitochondrial outer membrane and/or the label remained in the extramitochondrial space (n_e_). Since H_2_O can enter mitochondria, the total ^3^H radioactivity is the sum of that included in the mitochondria and that present outside the mitochondria.

A procedure must be followed by which the nmol taken up by mitochondria can be calculated.

The nmol of labeled substrate found in the pellet ntot are equal to n_i_ + n_e_ and therefore:

n_i_ = n_tot_ − n_e_

n_tot_ = ^14^Ccpm/SA

where ^14^Ccpm is the counts/min of each sample, as measured with a radioactivity counter and SA is the specific activity measured as ^14^Ccpm/nmol. This value can be calculated by measuring the radioactivity of a sample in which a known number of nmol of labeled substrate is added.

Therefore:

n_i_ = ^14^Ccpm/SA − ^3^Hcpm/SA_H2O_ × C_e_ − V_i_C_e_

Thus, to calculate n_i_, n_e_ must be measured.

The nmol taken up by mitochondria are essentially negligible with respect to those present in the initial suspension at the substrate concentration (C_e_). (For instance, if the substrate concentration C_e_ added in the Eppendorf cup is 1 mM or 1000 nmol/mL and since usually the taken-up substrate in nmol is <5 nmol, C_e_ can be considered unchanged). Therefore, n_e_ can be measured as the product of V_e_ × C_e_ where V_e_ is the volume of water outside mitochondria. Obviously, V_e_ = V_tot_ − V_i_ where V_tot_ is the volume of the pellet including ^3^H_2_O inside mitochondria and the volume of ^3^H_2_O outside mitochondria and V_i_ is the volume of water inside mitochondria.

Thus:

n_e_ = (V_tot_ − V_i_) × C_e_; n_e_ = V_tot_ × C_e_ − V_i_ × C_e_

V_tot_ can be obtained by measuring the radioactivity of ^3^H_2_O/SA_H2O_ where SA_H2O_ is the specific activity of the labeled water expressed as cpm of ^3^H_2_O/μL which can be measured from the cpm of ^3^H_2_O added in 1 mL of the medium in the Eppendorf cup.

Therefore:

n_i_ = ^14^Ccpm/SA − ^3^Hcpm/SA_H2O_ × C_e_ − V_i_C_e_

As a reminder, while the experiment described above is carried out under: 

Condition A: Substrate → Inhibitor

in a parallel experiment, carried out simultaneously, the inhibitor is added before the labeled substrate such that the radioactivity remained outside of the mitochondria (perhaps with some label bound to the outer membrane)

Condition B: Inhibitor → Substrates

Hence, the taken-up substrate in nmol can be calculated as the difference between condition A and condition B measurements:

n_i_ = ^14^Ccpm/SA − ^3^Hcpm/SA_H2O_ × C_e_ − V_i_C_e_ (under A condition) −

n_i_ = ^14^Ccpm/SA − ^3^Hcpm/SA_H2O_ × C_e_ − V_i_C_e_ (under B condition).

Importantly, V_i_ × C_e_, which is constant under both conditions, will be eliminated.

Notably, fluctuations that may result from the use of separate mitochondrial preparations can be eliminated when an experiment runs using the same preparation to include simultaneous measurements under both conditions A and B. Moreover, increasing the number of samples (3 or more) should allow the results to be expressed as mean +/− standard error.

Measurements can be made of the radioactivity of the ^14^C-substrate loaded mitochondria to monitor the exchange between an externally added metabolite/compound and the labeled metabolite/compound present in mitochondria after loading. In this case, the exported mitochondrial labeled metabolite/compound will be obtained as the difference of the radioactivity measured under condition B—the radioactivity measured under condition A.

Regrettably, isotopic techniques cannot distinguish between compounds that are taken up and similar compounds that are newly metabolized intramitochondrially from the portion of the taken-up substrate that is insensitive to penetrant inhibitors. Thus, the lack of a significant radioactivity accumulation in mitochondria due to the antiport between two labelled compounds could obscure the existence of several transport processes. For instance, in isotopic experiments, FUM was considered to be a non-penetrant anion due to an antiport that occurs between externally added FUM and malate, such that newly synthesized malate intramitochondrially from taken up FUM was exported to the extramitochondrial phase (see ref. [9]). Although the use of labeled substrates was fundamental in discovering and investigating in some detail metabolite transport, the necessity to impair mitochondrial metabolism makes mitochondria just a passive recipient of the incoming substrate without any ability to control or modulate intramitochondrial metabolism.

Independently of the techniques used when studying transport via a dynamic approach, since carrier proteins behave as enzymes in catalyzing metabolite movement across the mitochondrial membrane, any carrier-mediated transport could be characterized according to several criteria: the occurrence of saturation kinetics, the pH and temperature dependence, substrate specificity, the sensitivity to non-penetrant compound/s, the occurrence of antiport stoichiometry, the energy dependence, etc. Accordingly, any difference found among the investigated features should indicate the existence of separate transport mechanisms and consequently, separate carriers. It should be noted that since the traffic of metabolites can be derived from the combined action of two or more translocators (see below), this point must be taken into consideration when studying physiological metabolite movement.

Notice that V_max_, K_m_, and K_i_ values have only relative significance here since they are dependent on a variety of parameters essentially linked to medium composition (buffer, ionic strength, nature of ions, pH, etc.). However, they can be used in comparative studies.

Along with isotopic techniques, the stop inhibitor method can be applied to any analytical technique used to measure take up or efflux of substrate including for instance HPLC.

### 2.3. Final Comments

In the last 60 years, the mitochondrial transport has been investigated both via a dynamic and a static approach. We confirm that to study the role of the mitochondrial carrier in energy metabolism the experimental approaches described above are preferable.

Finally, we stress that the application of sophisticated techniques to study transport and, in particular, metabolism appears to be expensive and not simple to be used in the biochemistry laboratory: for instance, the conclusion that L-LAC addition to cancer cells can contribute to lipid synthesis proposed by Chen et al. [30] using high-resolution mass spectroscopy and transmission electron microscopy was a finding already published using essentially spectroscopic techniques: Chen et al. claim that their findings “demonstrate a link between lactate metabolism and the mitochondria of fermenting mammalian cell” however such a proposal was made 4 years before by showing that L-LAC addition to Hep G2 cell homogenates containing intact and coupled mitochondria resulted in the appearance of citrate, the precursor of fatty acid synthesis, in the extramitochondrial phase [21].

## 3. Cytosolic NADH Oxidation via the Mitochondrial Shuttles

**Figure 4:**
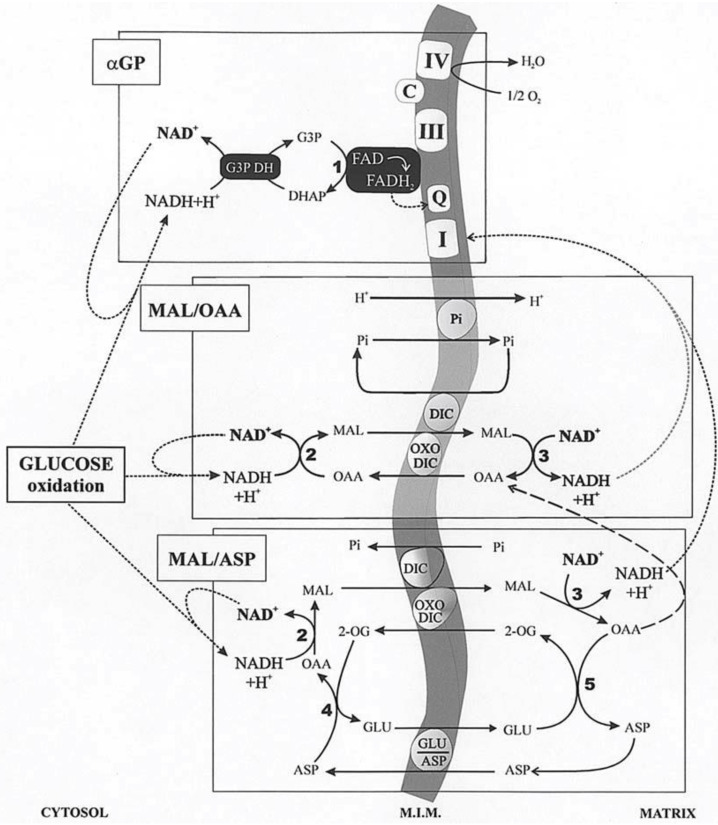
αGP, MAL/ASP, and MAL/OAA shuttles. MIM, mitochondrial inner membrane. Mitochondrial translocators: Pi, phosphate carrier; DIC, dicarboxylate translocator; OXODIC, oxodicarboxylate translocator; GLU/ASP, glutamate/aspartate translocator. Enzymes: 1, mitochondrial GPDH; 2 and 3, cytosolic and mitochondrial MDH respectively; 4 and 5, cytosolic and mitochondrial AAT respectively. From Atlante et al. [32] and Passarella et al. [12]. See text for details.

In glycolysis, NADH is formed in the reaction catalyzed by the glyceraldehyde-3-phosphate dehydrogenase. Given that NADH cannot enter mitochondria, its oxidation can occur both via L-lactate dehydrogenase (L-LDH) in the cytosol, with L-LAC production from PYR (with 2 ATP formed in substrate-level phosphorylation), and via the mitochondrial shuttles (with more than 30 ATP formed in the oxidative phosphorylation). A mitochondrial shuttle is a process in which enzymes and mitochondrial carriers transfer into or out of mitochondria molecules and/or charges that do not cross the mitochondrial inner membrane via simple diffusion. Important shuttles transfer reducing equivalents formed in glycolysis, where NADH is oxidized in the cellular cytosol by specific dehydrogenases and where the resulting reduced metabolites are then shuttled into the mitochondria. Then, specific mitochondrial enzymes re-oxidize these metabolites with concomitant reduction in mitochondrial cofactors (FAD/NAD^+^). These cofactors are then oxidized in the mitochondrial respiratory chain as electrons flow to oxygen, generating the electrochemical proton gradient used in the oxidative phosphorylation process that forms ATP. Finally, either directly, with no transport, in the case of the glycerol-3-phosphate/dihydroxy-aceton-phosphate (G3P/DHAP) shuttle, or indirectly, in a process that utilizes carrier-mediated transport, oxidized compounds are returned to the cytosol for the further reaction involved with NADH. The mitochondrial shuttles which allow for cytosolic NADH oxidation have already been reported in [9]. These are the malate/oxaloacetate (MAL/OAA), the malate/aspartate (MAL/ASP), the glycerolphosphate/dihydroxyacetone-phosphate (G3P/DHAP), the proline/glutamate (PRO/GLU) and the L-lactate/pyruvate (L-LAC/PYR). Obviously, the single contribution of each shuttle, as well as its reversibility, depends on the cell type in which they occur. MAL/OAA, MAL/ASP, G3P/DHAP, and L-LAC/PYR shuttles are involved in glycolysis. We will consider essentially the first three described in Figure 4. Demonstration of these shuttles in vitro is relatively simple by measuring photometrically the oxidation of externally added NADH to intact coupled mitochondria in the presence of the specific substrate and enzyme. In the case of the G3P/DHAP shuttle, Dawson and Cooney [31] using RKM showed that “the mitochondrial preparation oxidized added NADH even in the absence of further additions. However, this oxidation was strongly inhibited by rotenone, suggesting that damaged mitochondria were responsible for it. With NADH oxidation thus lowered, first glycerolphosphate dehydrogenase (G3PDH) and then glycerolphosphate G3P were added. The addition of the enzyme did not affect the NADH oxidation rate, but the introduction of glycerol-phosphate brought about an immediate and marked increase in the oxidation rate. This increase was abolished by cyanide, indicating its dependence on the mitochondrial respiratory chain”.

The malate/oxaloacetate shuttle (MAL/OAA) shuttle has already been proposed in 1960 by Klingenberg and Bucher [33]. It invoked the mitochondrial export of OAA in exchange for uptake of malate. Many researchers considered this alternative improbable, as the OAA concentration calculated from the equilibrium constant of the MDH reaction was very low and because the mitochondrial membrane was (erroneously) thought at the time to be impermeable to OAA. Indeed, a first indication of the permeability of mitochondria to OAA was offered by Haslam and Krebs [16] in 1968, later Gimpel et al. [34] suggested that OAA was transported in RLM via the dicarboxylate carrier. The in vitro transport of OAA in isolated RLM was later shown by checking the OAA capability to cause efflux of labeled dicarboxylates from loaded mitochondria [35,36]. In this case, the millimolar OAA concentration, a very high concentration compared to the micromolar physiological one, poses some doubts about the possible occurrence of OAA traffic across the mitochondrial membrane in vivo. Nonetheless, the use of the dicarboxylate carrier in OAA transport was ruled out since Pi efflux, due to externally added OAA, considered as an indication of OAA transport via the dicarboxylate carrier, proved to be lacking when malate was removed by using malic enzyme and NADP^+^ outside mitochondria. Surprisingly enough, Darvey [37] proposed that OAA can use the dicarboxylate and the Pi carrier to enter mitochondria. In another study, the authors used micromolar OAA concentrations, such as those measured in the cytosol. The capability of externally added OAA (at concentrations such as those measured in the cytosol) to bring about malate efflux from rat heart mitochondria was shown, as described above, by using the malate detecting system [38]. This was the first indication for a possible occurrence of a MAL/OAA shuttle used to export reducing equivalents from mitochondria. The mechanism of OAA efflux from mitochondria has been investigated because of its possible role in transferring cytosolic reducing equivalents into mitochondria. Consequently, reconstruction of the malate/OAA shuttle was shown made in a variety of mitochondria including those isolated from rat brain [39], rat kidney [40], rat cerebellar granule cells [41] saccharomyces cerevisiae [42], durum wheat and potato cells [43], and heart left ventricle [32]. For instance, the MAL/OAA shuttle was reconstructed by using non-synaptosomal rat brain mitochondria (RBM). The results demonstrated the occurrence of a carrier-mediated transport for OAA, sensitive to dicarboxylate analogs and mersalyl, and of the two isoenzymes of MDH. Malate/OAA shuttle rate appears to depend on the activity of the malate/OAA exchange across the mitochondrial membrane. This shuttle could account for the oxygen uptake by brain slices still occurring (40% of the control) when the transaminase which plays a major role in the MAL/ASP was 90% inhibited [39]. Interestingly, Pastore et al. [43] used plant mitochondria and showed that no NAD(P)H oxidation occurred arising from the MAL/ASP and the G3P/DHAP shuttles, and that NADH is oxidized in the presence of MAL and MDH, according to the presence of the MAL/OAA shuttle.

To date, the occurrence of the MAL/OAA shuttle is essentially ignored in all the biochemistry textbooks despite its in vitro reconstruction can be achieved in a 2-min experiment [44]. Unfortunately, no mention of either MAL/OAA or L-lactate/pyruvate (L-LAC/PYR) shuttles (see below) could be found in a paper dealing with pyridine nucleotide regulation of cardiac intermediary metabolism [45]. A study of metabolism in cancer cells metabolism reported that MAL/ASP shuttle “exerts control over NAD^+^/NADH homeostasis to maintain the activity of mitochondrial lactate dehydrogenase and to enable aerobic oxidation of glycolytic L-lactate in mitochondria”. However, again either MAL/OAA or LAC/PYR shuttles are not taken into consideration [46]. Again, the MAL/OAA shuttle, the malate cycle in terms of Klingenberg and Buecher [33], is not considered in a recent review by Borst [47], who commented about the malate cycle, but ignored all the findings described in other papers [9,44].

Which is the contribution of these three shuttles in any given type of mitochondria? Atlante et al. [32] described in detail the role played by the MAL/OAA shuttle in oxidizing external NADH (with comparison made with to the MAL/ASP and G3P/DHAP shuttles) in left heart ventricle mitochondria (RHLVM). This issue was addressed by reconstructing appropriate mitochondrial shuttles in vitro; here, use was made of RHLVM of both normotensive (WKY) and spontaneously hypertensive (SHR) rats at 5 and 24 weeks of age; they were used as model systems for left ventricle normotrophy and hypertrophy/hypertension, respectively.

To monitor the activity of the different shuttles, a comparison was made of NADH oxidation between mitochondria isolated from SHR and WKY rats.

**Figure 5:**
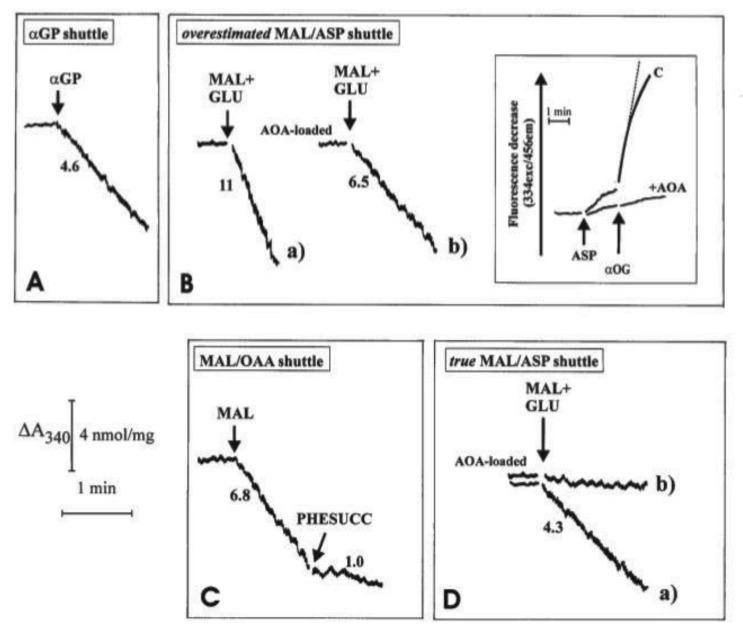
Oxidation of extramitochondrial NADH by WKY5-RHLVM. Reconstruction of the aGP shuttle (**A**), of the overstimated malate/aspartate (MAL/ASP) shuttle (**B**), of the malate/oxaloacetate (MAL/OAA) (**C**), of the true MAL/ASP shuttle (**D**). From [12,32], with permission from Arcane Editrice, Rome and Spandidos Publications, 2021.

How the three investigated shuttles have been reconstructed in vitro is shown in some detail in Figure 5. In all cases, mitochondria were initially incubated with NADH (0.2 mM) and the absorbance at 340 nm was monitored for a short period of time; the constancy of absorbance at 340 nm demonstrated that the internal mitochondrial Complex I was inaccessible to NADH, i.e., the intactness of mitochondria. Then, the appropriate substrates and enzymes were added to reconstruct either the α-GP or the MAL/ASP shuttles and to establish the occurrence of the malate/OAA shuttle.

Unloaded (A, B(a) and C) or mitochondria loaded with aminooxyacetate (AOA), an amino-transferase inhibitor which can enter mitochondria were incubated at 25 °C in 2.0 mL of standard medium, consisting of 0.2 M sucrose, 10 mM KCl, 20 mM HEPES-Tris, pH 7.2 and 1 mM MgCl_2_, plus 0.2 mM NADH in the presence of α-glycerolphosphate dehydrogenase (α-GPDH, 0.5 e.u.) (A), malate dehydrogenase (MDH, 2 e.u.) (C), α-oxoglutarate (0.1 mM) plus MDH plus aspartate aminotransferase (AAT, 1 e.u.) (B and D). Where indicated, additions were as follows: 1 mM α-glycerolphosphate (α-GP), 1 mM glutamate (GLU), 1 mM malate (MAL), 10 mM phenylsuccinate, (PHESUCC) a non-penetrant compound known to inhibit both the dicarboxylate and the oxodicarboxylate carriers. Confirmation was made that in AOA-loaded mitochondria AAT was strongly inhibited (B, inset); in fact, RHLVM, unloaded (C) or AOA-loaded mitochondria, suspended in 2 mL of standard medium in the presence of 2 µg rotenone and 1 mM sodium arsenite, were added with aspartate (ASP, 12 mM) and, after 1 min, with α-oxoglutarate (α-OG, 3 mM). The rate of decrease in fluorescence (λ_ex_ = 334 nm/λ_em_ = 456 nm) was then recorded and taken as a measurement of intramitochondrial AAT activity. Numbers along curves are rates of change in absorbance at 340 nm measured as tangents to the initial part of the progress curves and expressed as nmol NADH oxidized/min × mg mitochondrial protein.

In all cases, the operation of the shuttle was monitored continuously as a decrease with time of the NADH absorbance at 340 nm arising from metabolite interaction with the mitochondrial α−glycerol-phosphate dehydrogenase (m-GPDH), in the case of αGP/DHAP shuttle, or from traffic across the mitochondrial membrane (in the case of the other two shuttles) as revealed using specific substrate detecting systems (see Table 2). In Figure 5A, 1 e.u. α-GPDH was added to mitochondria. The DHAP concentration in the extramitochondrial phase was negligible since no change in absorbance occurred. As a result of the addition of 1 mM α-GP, a decrease in absorbance was observed at a rate equal to 4.6 nmol NADH oxidized/min × mg mitochondrial protein. The explanation for this finding (Figure 5A) is that added α-GP is oxidized by mitochondria in the reaction catalyzed by mitochondrial α-glycerolphosphate dehydrogenase (m-GPDH) located in the outer phase of the inner mitochondrial membrane and the product DHAP, in turn, oxidizes NADH outside the mitochondria in the reaction catalyzed by the added α-GPDH. Initial attempts to measure NADH oxidation as a result of the operation of the MAL/ASP shuttle were carried out as follows: mitochondria were incubated with the aspartate detecting system (ASP D.S.), consisting of 0.2 mM NADH, 0.1 mM α−ketoglutarate (α-KG), 0.5 e.u. malate dehydrogenase (MDH) plus 1 e.u. aspartate aminotransferase (AAT). Then, malate plus glutamate (1 mM each) were added to the sample and the change in absorbance was measured (Figure 5B). In this case, efflux of aspartate from the mitochondria was followed by its transamination via AAT to OAA, which in turn is reduced to malate in a reaction catalyzed by MDH outside mitochondria. The rate of decrease in absorbance was equivalent to 11 nmol NADH oxidized/min × mg mitochondrial protein (Figure 5B(a)). It must be emphasized that this is a considerable overestimate of the true activity of the shuttle as demonstrated by experiments using aminooxyacetate (AOA), (an AAT inhibitor which can enter mitochondria): first, it was confirmed that loading of mitochondria with AOA resulted in, essentially, a complete inhibition of mitochondrial AAT activity (Figure 5B, inset): when aspartate and α-ketoglutarate, the transaminase substrate pair, were added to “control” mitochondria a fast decrease in the fluorescence of the intramitochondrial pyridine nucleotides occurred. This decrease occurred because of OAA formation inside the mitochondria via mitochondrial AAT and its reduction to malate via MDH.

When using mitochondria loaded with AOA, no significant change in fluorescence was observed, indicating a complete inhibition of mitochondrial AAT. Given the mode of operation of the MAL/ASP shuttle, the shuttle should not occur in AOA-loaded mitochondria. However, when AOA-loaded mitochondria were used, the rate of NADH oxidation only decreased by approximately 40% to 6.5 nmol NADH oxidized/min × mg mitochondrial protein (Figure 5B(b)). This residual activity cannot be due to the MAL/ASP shuttle operation, which requires transamination in the mitochondrial matrix, and the true shuttle activity must be represented by the difference between the values in Figure 5B(a,b), i.e., 4.5 nmol NADH oxidized/min × mg mitochondrial protein (see below). One possible explanation for the residual activity is in Figure 5B(b) is that an MAL/OAA shuttle exists; this would be consistent with the permeability of heart mitochondria to OAA [9]. Direct evidence for this hypothesis has been obtained (Figure 5C): when mitochondria were incubated with MDH, (0.5 e.u.) and NADH alone, i.e., the OAA detecting system (OAA D.S.), no change in absorbance occurred showing that the OAA concentration in the extramitochondrial phase was negligible. Externally added 1 mM malate (MAL) caused oxidation of NADH at a rate of approximately 6.8 nmol/min × mg protein, indicative of the appearance of OAA outside the mitochondria. NADH oxidation was strongly inhibited (~85%) by 10 mM phenylsuccinate. The explanation of these findings is the following: MAL can enter mitochondria in exchange with endogenous phosphate or dicarboxylates; once inside the matrix, MAL is oxidized by mitochondrial MDH to OAA, which in turn can exit in a manner sensitive to phenylsuccinate. Once outside mitochondria, it is reduced by NADH in the presence of MDH in a reconstructed MAL/OAA shuttle.

Based on these findings, indicating that the activity of the MAL/ASP shuttle, as shown in Figure 5B(a) and previously by others is overestimated, being dependent on the efflux of both aspartate, which is converted to OAA via transamination, and OAA itself, a new procedure was developed to continuously monitor MAL/ASP shuttle activity. Here, ASP D.S was added to the mitochondrial suspension in the sample cuvette, whereas ASP D.S. without AAT was added to the reference cuvette. Then, malate plus glutamate (1 mM each) were added simultaneously to both the sample and reference cuvettes and the oxidation of NADH was recorded. In the sample cuvette (CUV 1) OAA derives from a simple exchange and because of the aspartate transamination, while in the reference cuvette (CUV 2) only OAA efflux can be monitored. The absorbance difference between that of CUV 1 and CUV 2 gives the true rate of MAL/ASP shuttle, which was found to be 4.3 nmol NAD(P)H oxidized/min × mg mitochondrial protein (Figure 5D(a)). Consistently, when using AOA-loaded mitochondria, the activity of the shuttle was effectively abolished (Figure 5D(b)). As expected, the measured true activity of the MAL/ASP shuttle was very close to the difference between the activities shown in Figure 5B(a,b).

**Figure 6:**
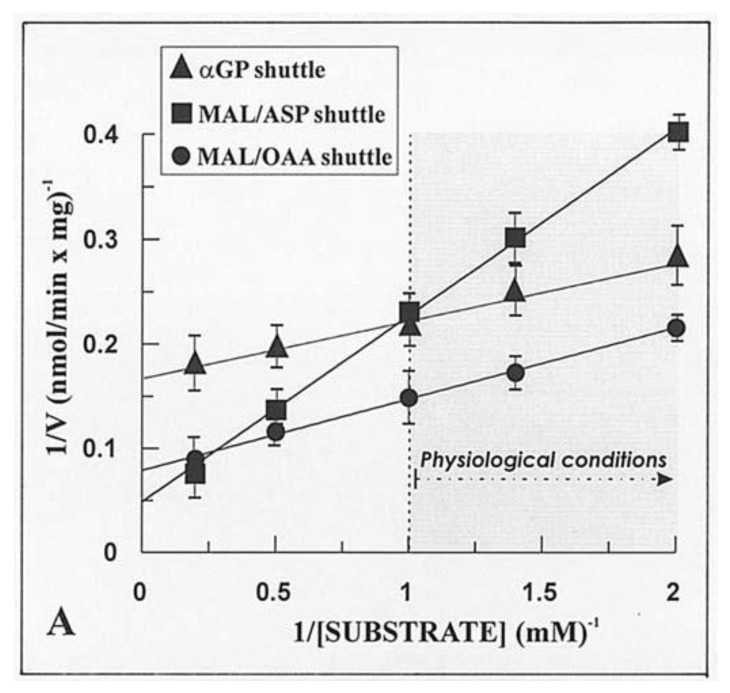
The dependence of the rate of NADH oxidation via the three shuttles on increasing substrate concentrations. From [12,32], with permission from Arcane Editrice, Rome and Spandidos Publications, 2021.

Given that the real contribution of the investigated shuttles depends also on the physiological concentration of the substrates, the dependence of the rate of decrease in absorbance of NADH, i.e., the rate of the in vitro reconstructed shuttles was investigated as a function of increasing concentrations of either α-GP or MAL. Hyperbolic reaction kinetics were found for the α-GP/DHAP MAL/ASP and MAL/OAA shuttles, and Figure 6 shows a typical double reciprocal plot for data obtained with WKY5-M. Km values, i.e., the substrate concentration, which gives half the maximum rate (Vmax), and Vmax values were determined in several experiments carried out with different mitochondrial preparations. The values obtained with SHR5-M, SHR24-M, WKY5-M, and WKY24-M were calculated along with statistical analysis using the ANOVA test. In five experiments, the Vmax values of the MAL/ASP shuttle as measured in SHR5 vs. SHR24 differed significantly both from one another and from the same-age WHY samples (*p* < 0.05). In contrast, no statistically significant differences were found for Vmax values of the α-GP/DHAP shuttle independently of the age and hypertrophy/hypertension states.

Interestingly, in this case, the rate of NADH oxidation was shown to depend on the rate of MAL/OAA antiport across the mitochondrial membrane. This raises the question as to whether the metabolite transport processes play a role in metabolic regulation.

In conclusion, most NADH oxidation occurs via the MAL/OAA shuttle, the activity of which increases with time and with the progression of hypertrophy and development of hypertension as judged by statistical ANOVA analysis. In contrast, the other two shuttles investigated were shown to make only a minor contribution to NADH oxidation in a manner essentially independent of age and progression of hypertrophy/hypertension. The rate of NADH oxidation via the MAL/OAA shuttle is the rate of malate transport in exchange with OAA. Therefore, the contribution of the MAL/OAA shuttle to oxidation of NADH is higher than those of the MAL/ASP and α-GP shuttles, even if OAA cannot enter mitochondria.

It is somewhat surprising that in various papers dealing with the oxidation of cytosolic NADH, the occurrence of the MAL/ASP shuttle has been considered without any experiment carried out to ascertain the OAA efflux. For instance, in a paper by Gregory et al. [48], dealing with reducing equivalent transfer to the mitochondria during gluconeogenesis and ureagenesis, the MAL/OAA shuttle was completely ignored. The assumption that OAA cannot cross the mitochondrial membrane drove the conclusion that “the bulk of oxaloacetate, formed by PYR carboxylation within the mitochondria during gluconeogenesis from lactate, is transported to cytoplasm as aspartate” [49].

In a paper aimed to investigate the influence of thyroid hormone on the NADH shuttles in cardiac and liver mitochondria, MAL/ASP and α-GP/DHAP shuttle capacities were significantly increased in cardiac mitochondria from adult rats treated for 9 days with T3 compared to saline-treated controls, but a possible role for the MAL/OAA shuttle was ignored [50]. In another paper by the same group, it was suggested that “sufficient malate/aspartate and α-glycerophosphate shuttle capacity exists in cardiac mitochondria to accommodate increased shuttle flux as hypertrophied myocardium becomes more glycolytically active” [51].

We believe that any study of the MAL/ASP shuttle should be accompanied by experiments in which the occurrence of the MAL/OAA shuttle is also investigated. For instance, it should be noted that in a study where MAL/ASP shuttle was investigated in vascular smooth muscle [52] in the presence of AOA, only 20% inhibition of glucose oxidation occurred. This poses the question as to whether other shuttles, not involving transaminase reactions, can contribute to glucose oxidation in the heart as such as/OAA and L-LAC/PYR shuttles (see below).

## 4. Phosphoenolpyruvate (PEP) Transport in Mitochondria

In the last century, PEP transport in and from mitochondria has been poorly investigated. The first evidence of PEP capability to cross the mitochondrial membrane was provided in 1967 by Gamble and Mazur who showed that citrate addition to rabbit liver mitochondria (RbLM) resulted in the PEP appearance outside mitochondria [53]. The authors concluded that “This could be due to a specific positioning or compartmentation of the reaction or, alternatively, to a specific permeability of the membrane”. As reported in [9], Drahota et al. [54] and Wiese et al. [55] showed that PEP can enter mitochondria. A revolution in PEP mitochondrial transport and metabolism occurred in the first decade of the third millennium, when PEP transport in mitochondria was investigated, with evidence of the existence of the mitochondrial PK both in plant [56] and mammalian mitochondria [57,58].

In the first case, coupled mitochondria isolated from Jerusalem artichoke tubers (JAM) were used and an investigation was made utilizing mainly the dynamic approach. Interestingly, as opposed to the expected increase in in NAD(P)H fluorescence, (an increase due to the NAD(P)^+^ reduction arising from the via intramitochondrial metabolism of the newly synthesized PYR via a mitochondrial by PK), upon PEP addition to JAM, intramitochondrial NAD(P)H oxidation was found due to the activation of the alternative oxidase (AOX) determined by the capability of the AOX inhibitors, both propyl-Gallate and salicyl-hydroxamic acid (SHAM), AOX inhibitors, to inhibit this process. Nonetheless, in spite of the four steps involved in this process, i.e., PEP uptake, the PK reaction, AOX activation by PEP and pyruvate with the consequent intramitochondrial NAD(P)H oxidation, the decrease in fluorescence proved to mirror the PEP uptake as shown by applying control strength criterion.

It was found that:

- PEP can be metabolized by JAM by virtue of the presence of the mitochondrial PK, shown both immunologically and functionally, located in the inner mitochondrial compartments, and distinct from the cytosolic PK (shown by the different pH and inhibition profiles).
- PEP uptake into JAM occurs in a proton compensated manner, in a carrier-mediated process.
- The PEP addition resulted in PYR and ATP production, as monitored via HPLC, with their efflux into the extramitochondrial phase as detected fluorimetrically (see above). Such an efflux occurs via the putative PEP/PYR and PEP/ATP antiporters that differ from each other and from the PYR and the adenine nucleotide carriers, based on the different sensitivities to non-penetrant compounds. These carriers were shown to regulate the rate of efflux of both and ATP. The appearance of citrate and OAA outside mitochondria was also found because of PEP addition.

The PK existence in mitochondria was confirmed in pig liver mitochondria [57,58]. The researchers took advantage of a bioinformatic suggestion: as a result of a search for ‘‘mitochondrial pyruvate kinase” in the NCBI genome database, a gene (LOC100154270) coding for a protein similar to PK which that is predicted to have a mitochondrial localization by Target P 1.1 analysis (reliability class = 3) was found in the Sus scrofa genome database. However, the PK occurrence was shown by monitoring photometrically the PK reaction in solubilized mitochondria with either PEP or ADP used as a substrate. In distinction with the cytosolic isoenzyme, the mitochondrial PK showed a sigmoidal dependence on either PEP or ADP concentrations. The occurrence of the mitochondrial PK was confirmed by immunological analysis. Titration with digitonin showed that mPK is restricted to the matrix. PEP addition to mitochondria that resulted in a reduction in the intramitochondrial NAD(P)^+^ was inhibited by either the non-penetrant thiol reagent mersalyl or by arsenite, an inhibitor of the pyruvate dehydrogenase complex.

Citrate/OAA appearance outside mitochondria also occurred as a result of PEP addition to PLM. Taken together, these findings support a role for PEP itself in triggering fatty acid synthesis via its mitochondrial metabolism. This process is described in the paper by Di Ciaula et al. [59].

These findings shone new light to previous results previously published in the Pasqualina Pierro Ph.D. thesis work (1991–1995) and only as reported in a meeting [60] and in [12]. We offer these experiments as a matter of discussion for further investigation.

**Figure 7:**
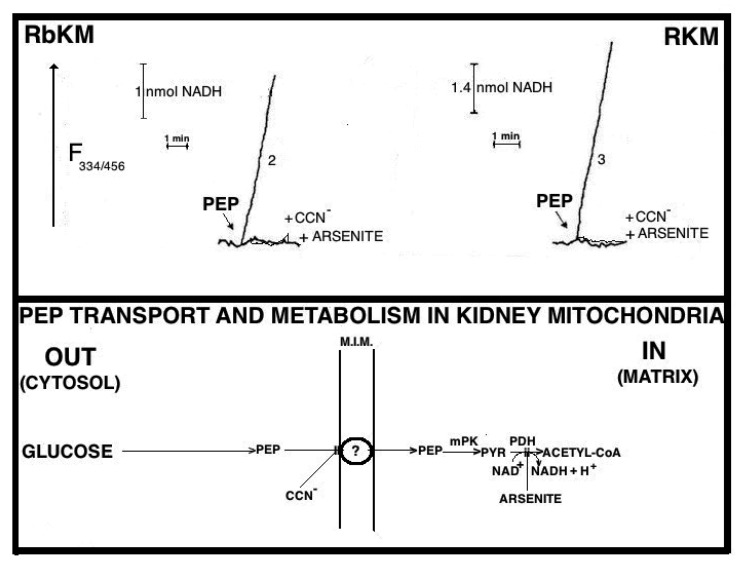
Phosphoenolpyruvate (PEP) can enter both rabbit and rat kidney mitochondria. From [12]. From Aracne Editrice with permission 2021. Fluorimetric investigation was made of the change in the redox state of the intramitochondrial pyridine nucleotides caused brought about by the addition of PEP. Rabbit and rat kidney mitochondria (RbKM and RKM, respectively) (2 mg protein) were suspended at 25 °C in 2 mL of standard medium consisting of 15 mM KCl, 1 mM MgCl_2_, 100 mM TRIS-HCl, pH 7.4. and the redox state of intramitochondrial pyridine nucleotides was followed fluorimetrically (λ_ex_ = 334 nm/λ_em_ = 456 nm) as a function of time. First, either RbKM or RKM were incubated for 3–5 min with FCCP (1.25 mM) and then rotenone was added (2 mg) (not shown). At the arrow, PEP (2.5 mM) was added either in the absence or presence of α-cyano-4-hydroxy-cynnamate (CCN^−^) or arsenite (0.01 and 1 mM, respectively). The rate of fluorescence increase, measured as the tangent to the initial part of the progress curve, is expressed as nmol of intramitochondrial NAD(P)^+^ reduced/min × mg protein. PEP uptake by mitochondria was prevented in the presence of either α-cyano-4-hydroxycynnamate (CCN^−^ 10 μM), or benzylmalonate (5 mM) (not shown), which can inhibit a variety of carriers. These simple experiments have produced the first evidence that mammalian mitochondria contain their own PK and showed that mitochondrial PEP transport occurs in a carrier-mediated manner.

Fluorimetric investigation of the change in the redox state of the intramitochondrial pyridine nucleotides brought about by the PEP addition was made (Figure 7). Mitochondria were isolated from rabbit kidney mitochondria (RbKM) and RKM that differ from one another with respect to the localization of the PEP carboxykinase, which is present in both cytosol and mitochondria of RbKM but is absent in RKM. In both cases, PEP proves to enter mitochondria as shown by the increase in the intramitochondrial NAD(P)H fluorescence. The addition of arsenite (1 mM), an inhibitor of the pyruvate dehydrogenase complex (PDH), resulted in complete inhibition of the NAD(P)H formation. This shows that the fluorescence increase is due to the oxidation via PDH of the PYR newly synthesized from imported PEP via the mitochondrial PK.

**Figure 8:**
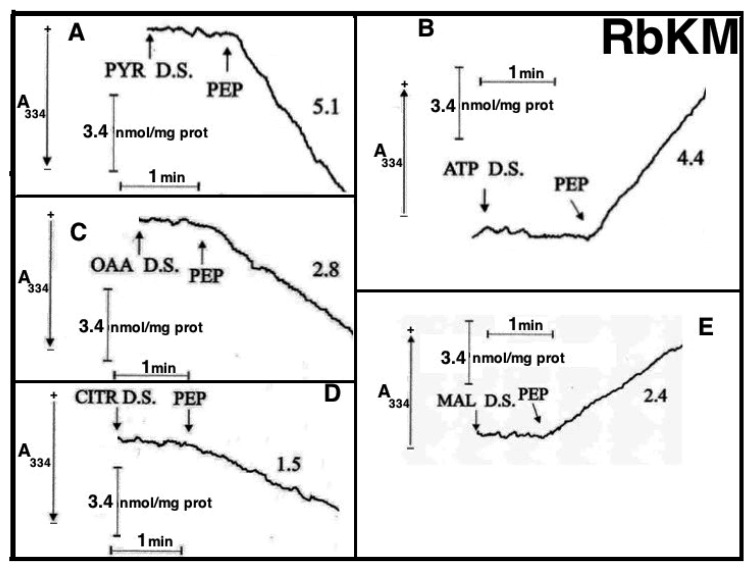

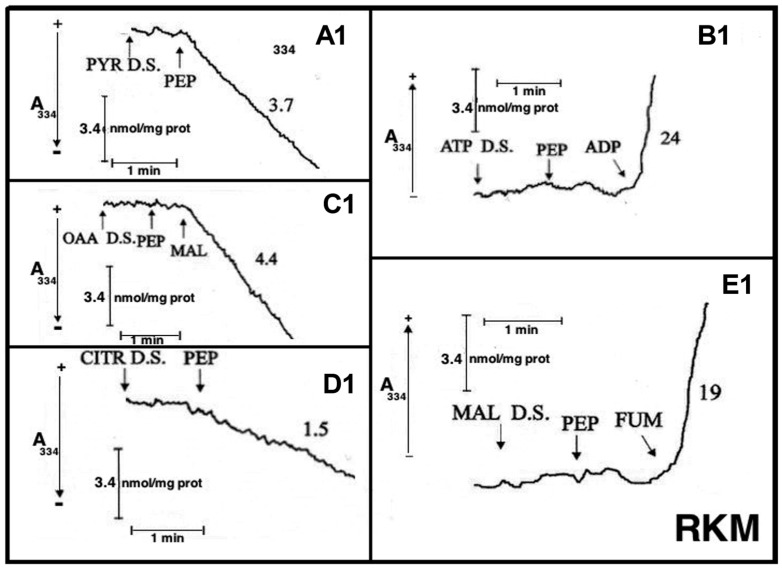
Appearance of pyruvate (PYR) (**A**), ATP (**B**), oxaloacetate (OAA) (**C**), citrate (CITR) (**D**), and malate (MAL) (**E**) in the extramitochondrial phase space induced by PEP addition to rabbit kidney mitochondria (RbKM) and appearance of PYR (**A1**), ATP (**B1**), OAA (**C1**), CITR (**D1**), and MAL (**E1**) in the extramitochondrial phase space induced by the addition of PEP, ADP, MAL, and fumarate (FUM) to rat kidney mitochondria (RKM). From [12]. With permission from Aracne Editrice, 2021.

At that time PK was considered only a cytosolic enzyme and no further investigation on this issue was pursued. However, in light of the metabolite transport paradigm [9], postulating that net carbon uptake by mitochondria is accompanied by the efflux of certain newly synthesized compounds, an investigation was made to ascertain whether or not the addition of PEP to RbKM and RKM could result in the efflux of pyruvate and ATP, the products of the mitochondrial PK reaction (Figure 8A,B). Either RbKM or RKM were suspended at 25 °C in 2 mL of standard medium consisting of 15 mM KCl, 1 mM MgCl_2_, 100 mM TRIS-HCl, pH 7.4 in the presence of either NADH (0.2 mM) (A and A1, C and C1, D and D1) or NAD(P)^+^ (0.2 mM) (B and B1, E and E1) and absorbance at 334 nm (A334) was continuously monitored.

At the times indicated by the arrows the pyruvate detecting system (PYR D.S.) (A, A1), the ATP detecting system (ATP D.S.) (B, B1), the OAA detecting system (OAA D.S.) (C, C1), the citrate detecting system (CITR D.S.) (D, D1), and the malate detecting system (MAL D.S.) (E, E1) were added followed by PEP (2.5 mM), (A–E, A1–E1) ADP (0.05 mM) plus P1,P5-di(adenosine-5′) pentaphosphate (Ap_5_A) (0.01 mM), added to inhibit the adenylate kinase (B, B1), MAL (1 mM) (C, C1) or FUM (1 mM) (E, E1).

In all cases, use was made of the metabolite/compound detecting systems described in Table 2**.** The rate of NADH oxidation/NADP^+^ reduction was measured as a tangent to the initial part of the progress curves and expressed as nmol NADH oxidized/NADP^+^ reduced/min × mg mitochondrial protein.

As a result of PEP (2.5 mM) addition to RKM both PYR and citrate were detected in the extramitochondrial space. This contrasted with what was found with RbKM, where no efflux of ATP, OAA, or malate was found, as shown by the constancy of the unchanged absorbance at 334 nm in the presence of their detecting systems. As a control, RKM was tested for their ability to export ATP, OAA, and malate upon the addition of ADP (0.05 mM), malate (1 mM), and fumarate (1 mM), most likely via the ADP/ATP, the MAL/OAA and the FUM/MAL antiporters, respectively.

Both, to gain further insight into the permeability of RKM to PEP and to investigate whether or not PEP can be synthesized in the mitochondrial matrix via PK and exported outside RKM, PYR (2.5 mM) was added to mitochondria. Since the appearance of PEP outside the mitochondria cannot be continuously monitored, neither via PK and L-LDH, due to the presence of PYR, nor as ATP formation via the ATP D.S., due to the presence of ADP, which can drive both ATP synthesis and its export, an enzymatic assay was made to ascertain the PEP presence. PEP was found in the supernatants (about 6 nmol/mg mitochondrial protein) with its amount increasing up to 16 nmol/mg mitochondrial protein due to the presence in the incubation medium of arsenite (1mM), which can prevent PYR oxidative decarboxylation via PDH, making more PYR available for PEP synthesis.

**Figure 9:**
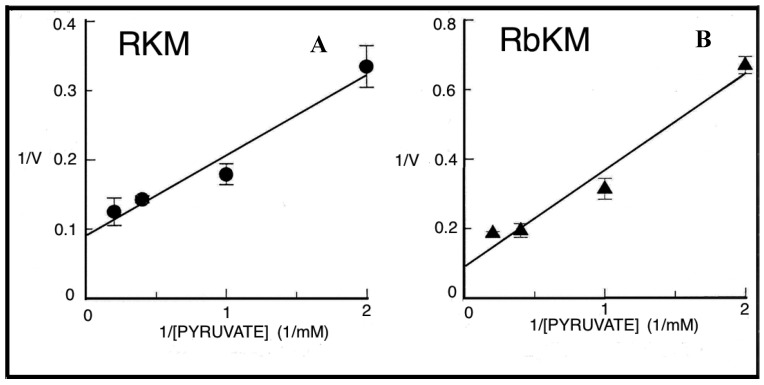
The dependence of the rate of the pyruvate/[^14^C]-phosphoenolpyruvate (PEP) exchanges in either rat (**A**) or rabbit (**B**) kidney mitochondria on the external pyruvate concentration. From [12]. With permission from Aracne Editrice, 2021. [^14^C]-PEP-loaded mitochondria (1 mg protein) were incubated at 4 °C in 1 mL of a standard medium consisting of 15 mM KCl, 1 mM MgCl_2_, 100 mM TRIS-HCl, pH 7.4 plus 1 µg of µg rotenone, 3 µg oligomycin in the presence of 5 mM arsenite and 10 mM oxalate. After 1 min incubation, the assay was started with pyruvate, at the concentrations indicated, and was stopped after 6 seconds by the addition of 10 mM phenylsuccinate. The exported ^14^C-PEP was assayed as reported above. Both RKM and RBKM were loaded with ^14^C-PEP and its efflux measured according to the stop inhibitor method.

**Figure 10:**
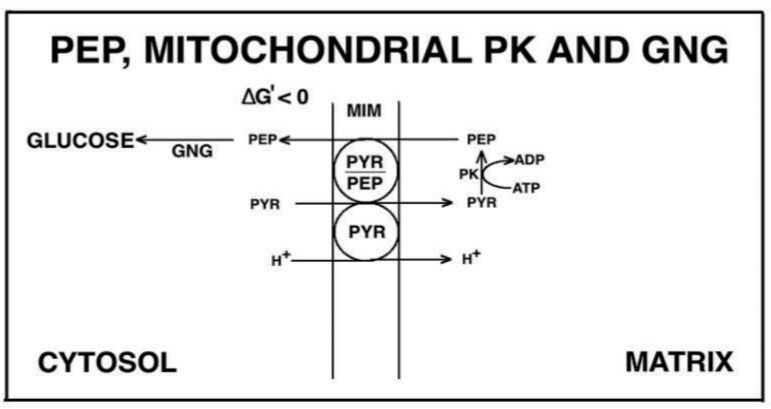
The role of the mitochondrial pyruvate kinase in gluconeogenesis in rat kidney. The following scenario is proposed: pyruvate (PYR) derived from L-lactate and amino-acids enters mitochondria via its own carrier; once inside the matrix via the mitochondrial pyruvate kinase (PK) phosphoenolpyruvate (PEP) is synthesized, the reaction being made possible by the removal of the newly synthesized PEP which is exported in exchange with PYR via the putative PYR/PEP antiporter.

To confirm the capability of externally added PYR to cause PEP efflux from mitochondria a different isotopic approach was required. Thus [^14^C]-PEP-loaded mitochondria were used. PYR was added at increasing concentration and after 1 min incubation, the mitochondrial suspension was centrifuged and the supernatants were assayed for [^14^C]-PEP presence. In both cases, PYR/^14^C-PEP exchange was found to occur with saturation kinetics as shown by studying the dependence of the rate of PEP efflux as a function of increasing PYR concentrations (Figure 9). Vmax values were about 9.5 and 7.3 nmol/min × mg protein, respectively and Km values were 0.85 and 1.4 mM for RKM and RbKM, respectively. Figure 10 describes the possible scenario derived from the above experiments.

The PEP-mitochondria affair remains open. However, it must be stressed that PEP transport and metabolism in mitochondria—as well as the mitochondrial PK—have not been given any consideration in several papers published in the second decade of this century. These include a paper by McCommis and Finck [61] paper dealing with mitochondrial PYR transport, one by Cerdan (2017) [62] in which twenty-seven years of cerebral PYR recycling is reported, and another by Chinopoulos [63], in which biochemical pathways in which twenty-seven of cerebral years biochemical pathways connecting glucose and other metabolites to PYR and L- or D-LAC are described. A revision of the PEP metabolism in the light of old and new achievements findings appears to be necessary.

Interestingly, in skeletal muscle, trioses are generated via reversal of PK and L-LAC contributes to glycerol and glycogen via reversal of PK [64].

## 5. L-Lactate Transport in Mitochondria

### 5.1. The L-Lactate History

Although the L-LAC history begins more than a century ago, the role of mitochondria in L-LAC metabolism has received recognition only at the outset of the third millennium. Notably, in the past two decades, authors writing the L-LAC history had different scientific backgrounds. Physiologists consider L-LAC as a major product of exercise, neuroscientists approach L-LAC as an important oxidative substrate for brain energy metabolism, while others are experts in mitochondrial research involved in the role of mitochondria in the L-LAC metabolism. The consideration of L-lactate biochemistry, when approached from different perspectives, explains why limited knowledge of mitochondrial bioenergetics would lead to potential misunderstandings (see ref. [7]).

**Figure 11:**
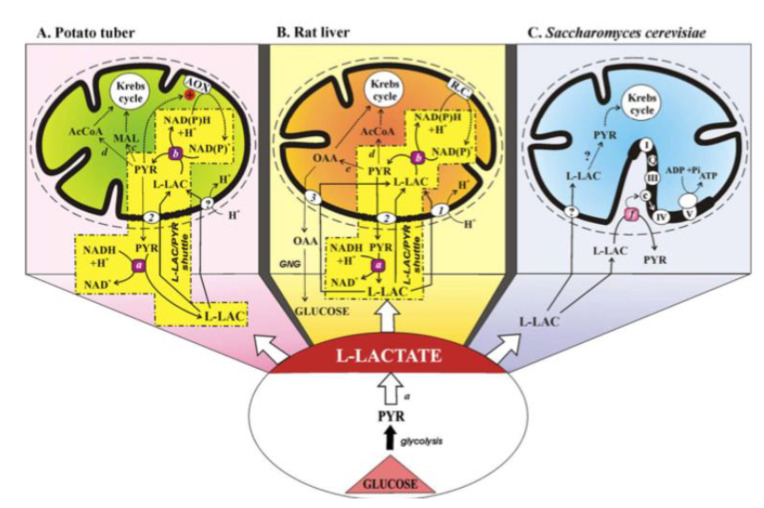
The mitochondrial metabolism of L-lactate. From Passarella et al. From [10]. With permission from John Wiley and Sons, 2021.

Until the mid-1980s, L-LAC was considered to be a waste product rather than an energy source for a variety of cells, and L-LAC was postulated to be taken up and oxidized (see [10]). Thereafter, the role of mitochondria in L-LAC metabolism has been demonstrated in spermatozoa, liver, heart, skeletal muscle, brain, plant, and yeast mitochondria. As for the latter, the history of the L-lactate–mitochondrial affair up to 2008 has been outlined in a mini-review by Passarella et al. [10]. Figure 11 describes the status of the L-lactate–mitochondria affair in 2008.

(A) The mitochondrial metabolism of L-lactate in potato tuber. The sequence of events involved in mitochondrial metabolism of L-lactate (L-LAC) is envisaged as: uptake into mitochondria of L-LAC, synthesized in the cytosol by anaerobic glycolysis, perhaps via the L-LAC/H^+^ symporter; oxidation of the L-LAC to PYR by the mL-LDH located in the inner mitochondrial compartment; activation of alternative oxidase (AOX) by the newly synthesized PYR, oxidation of the intramitochondrial NAD(P)H via AOX with efflux of PYR via a putative L-LAC/PYR antiporter and the oxidation of cytosolic NADH in a non-energy-competent L-LAC/PYR shuttle. PYR conversion could also occur to AcetylCoA and malate via pyruvate dehydrogenase and malic enzyme, respectively.

(B) The mitochondrial metabolism of L-lactate in the liver. Externally added L-LAC can enter RLM where it is oxidized by the mitochondrial L-LDH. L-lactate can cause efflux in the extramitochondrial phase of PYR and OAA newly synthesized in the mitochondrial matrix via mL-LDH and pyruvate carboxylase. The metabolite efflux occurs by virtue of the occurrence of three carriers for L-LAC transport in mitochondria: the L-LAC/H^+^ symporter and the L-LAC/PYR and L-LAC/OAA antiporters. The LAC/PYR antiporter accounts for the LAC/PYR shuttle which transfers reducing equivalents from the cytoplasm to the mitochondrial respiratory chain. The L-LAC/OAA antiporter accounts for novel gluconeogenesis. OAA and PYR (via the pyruvate dehydrogenase) could also fill up the Krebs cycle intermediate pool.

(C) The mitochondrial metabolism of L-lactate in Saccharomyces cerevisiae. Externally added L-LAC can enter mitochondria via a putative L-LAC/H^+^ symporter. In mitochondria, an NAD-dependent mL-LDH exists. Moreover, in the intermembrane space L-LAC is oxidized to PYR with reduction in cytochrome c by a flavin mitochondrial L-lactate:cytochrome c oxidoreductase in an energy competent manner. Abbreviations: AcCoA, acetyl-CoA; AOX, alternative oxidase; GNG, gluconeogenesis; L-LAC, L-lactate; MAL, malate; OAA, oxaloacetate; PYR, pyruvate; R.C., respiratory chain; transport and oxidation processes the existence of which has not yet been confirmed. Enzymes: a, cytosolic L-LDH; b, mitochondrial L-LDH; c, malic enzyme; d, pyruvate dehydrogenase; e, pyruvate carboxylase; f, L-lactate:cytochrome c oxidoreductase (Cyb2p). Mitochondrial carriers: 1, L-LAC/H^+^ symporter; 2, L-LAC/PYR antiporter; 3, L-LAC/OAA antiporter.

Interestingly, de Bari et al. [65] provided evidence for the presence of an intermembrane L-lactate oxidase that generates H_2_O_2_ sufficient to activate the known ROS response elements, signaling mitochondrial and other adaptations to exercise. That work provides a possible mechanism by which L-LAC generation in muscle exercise participates in the feedback loop. L-LAC generation in exercise leads to adaptations facilitating high rates of lactate disposal in exercise. It is worth noticing that the putative L-LAC oxidase could be a candidate to the new role proposed for L-LAC as “lactormone”, i.e., in Brooks’ term [66] as a cell-signaling molecule that is involved in the adaptive response to exercise. In 2015, Paventi, Lessard, Bailey, and Passarella [67] found that in boar sperm capacitation L-LAC, but not PYR can contribute to mitochondrial membrane potential increase as monitored via safranine fluorescence, this both strongly suggesting that L-LAC is the ultimate product of glycolysis and confirming that L-LAC must enter mitochondria to be oxidized by the mL-LDH (see also [10]).

We will consider some aspects of the mitochondrial L-LAC transport and metabolism in neuronal and cancer cells not already considered in [10].

### 5.2. The L-Lactate Mitochondrial Metabolism Plays a Major Role in Neuronal Cells

A better understanding of the role of L-LAC in neuronal energy metabolism and in particular in mitochondrial energy metabolism (see [10]) requires some attention. In fact, for almost a century this molecule was considered just a waste product of the brain during hypoxia. The lactic acidosis hypothesis of delayed neuronal damage post cerebral ischemia was proposed in 1981 [68], and the researchers quickly explained the cellular mechanism that leads to this phenomenon, i.e., the fall in pH post-ischemia leads to delayed neuronal damage and this drop is due to the ischemic production of L-LAC. Upon attempting to establish an in vitro model of cerebral ischemia by showing that lactic acidosis would worsen ischemic neuronal damage, the investigators were surprised to find that acidic pH provided slight protection, while L-LAC provided dramatically better protection [69].

Consequently, a seminal paper demonstrated the ability of neuronal tissue to utilize L-LAC as its sole oxidative energy substrate to maintain neuronal function [70]. This finding did not sit well with the prevailing dogma of the time regarding glycolysis, L-LAC, and oxidative energy metabolism leading to a long-lasting debate that has not subsided to this day. Nevertheless, the above-mentioned in vitro model system allowed the further exploration of the role of L-LAC as an oxidative substrate of neuronal tissue main substrate for oxidative energy metabolism. By 1994, Izumi et al. [71] confirmed this finding. Similarly, Larrabee [72,73] provided further support for neuronal oxidative utilization of L-LAC. L-LAC was shown to be the obligatory energy substrate for the recovery of neuronal function from hypoxic/ischemic insult [74,75]. Using in vivo recording in the rat brain, Hu and Wilson [76] demonstrated that fluctuations in the levels of extracellular L-LAC and oxygen levels are coupled to neuronal activity. An increase in L-LAC output upon neuronal excitation in vitro was shown to serve the need for increased energy demands of excited neurons [77]. In contrast, the blockade of L-LAC transport via the monocarboxylate transporter 1 (MCT1) exacerbates delayed neuronal damage in a rat model of cerebral ischemia [78]. This finding established L-LAC as the preferential oxidative energy substrate when ATP stores dwindle to the point that glycolytic glucose phosphorylation is unattainable and possibly even under normal physiological conditions. In 2003, Smith et al. [79] showed that L-LAC is the preferred fuel for human brain metabolism in vivo and Dalsgaard et al. [80] demonstrated that reduced cerebral metabolic ratio in exercise reflects L-LAC metabolism rather than accumulation in the human brain. Thus, in 2006 L-LAC was suggested to be the ultimate cerebral oxidative energy substrate [81]. By 2007, it was demonstrated that L-LAC, not PYR, is the neuronal aerobic glycolysis end product in vitro [81,82]. In that study relatively specific LDH inhibitors were used to inhibit either the L-LAC-to-PYR reaction or the PYR-to-L-LAC one, enabling the investigators to determine that L-LAC, rather than PYR, is the substrate that is being oxidized intra-mitochondrially by LDH.

During the preparation of the 2006 review [81], a thorough search of older literature from the 1920s and 1930s, rediscovered a throve of studies, most of which were performed by one group of biochemists. Those investigators demonstrated and were aware of the ability of brain tissue preparation to make lactic acid disappear in the presence of oxygen, a process the investigators interpreted as a mechanism of lactic acid clearance [83,84,85,86,87,88,89,90,91,92].

The prevailing dogma at the time had been that lactic acid is a waste product to be cleared. It is important to clarify that Krebs and Johnson were not sure about their suggestion that PYR is the mitochondrial substrate of the TCA cycle [93,94,95], but that suggestion was the one responsible for the decision by the elucidators of the glycolytic pathway to place PYR as its final aerobic product. This digging might suggest that biochemical archeology could still be a source of inspiration.

That L-LAC can be a neuronal energy source was also shown by O’Brien et al. [95] and Wyss et al. [96].

A short commentary [97] weighed in on the results published by Larsen et al. [98]. These investigators described their attempts to elucidate the mechanism behind the observed exercise-induced reduction in the cerebral metabolic ratio (CMR) as measured in healthy human subjects. They no longer used the accepted definition of CMR as O_2_/glucose, but rather focus instead on the more accurate definition of CMR, i.e., O_2_/(glucose + ½ L-LAC), to make their estimates. They clearly show that as their subjects approached the maximal work load and exhaustion under control conditions, the CMR [O_2_/(glucose + ½ L-LAC)] fell from the expected value of approximately 6 to about 3. When the same measurement was done for CMR (O_2_/glucose), its value did not change significantly throughout the experimental period. Concomitantly, the a–v differences for glucose and oxygen, even at the maximum workload, increased only slightly in comparison to the values at rest. In contrast, the a–v differences for total carbohydrates (CHO) at maximal workload were significantly higher than the values at rest, an increase that could be attributed almost entirely to the significantly higher a–v difference for L-LAC. Based on the results of Larsen and colleagues, Schurr concluded that L-LAC is a major and crucial player in the normal function of both brain and muscle [97]. In 2011, it was shown that aerobic production and utilization of L-LAC satisfy increased energy demands upon neuronal activation in hippocampal slices and provide neuroprotection against oxidative stress [99]. The authors analyzed, among others, the results of the study by Hu and Wilson [76] who measured glucose, L-LAC, and oxygen level in brain tissue in vivo during rest and stimulation. The analysis demonstrated that, during continuous stimulation, brain tissue consumes, oxidatively, gradually more L-LAC and less glucose, a process that allows for a more efficient ATP production to support neuronal activation induced by such stimulation. Moreover, in vitro experiments carried out by Schurr and Gozal [99] indicated that the production of reactive oxygen species (ROS) in response to the presence of the excitotoxic neurotransmitter, glutamate was reduced significantly when L-LAC was the sole energy substrate. PYR—as the sole energy substrate—could not reduce ROS production. Despite the continuous accumulation of studies supporting L-LAC’s major role in brain energy metabolism, doubts about their validity are persisting, even today. In a review [100] the authors opined on the persistence of doubt among scientists regarding L-LAC’s role as a major oxidative substrate for energy metabolism in the brain and elsewhere and offered that a “habit of mind” may explain the persistence of that doubt. Considering the proposed function of L-LAC in energy metabolism, the accuracy of current methods and techniques used to measure brain tissue metabolic rates of glucose only, not taking into account the contribution of L-LAC to these measurements was questioned.

Clearly, there is ample evidence to support the concept that brain L-LAC is the glycolytic end product, both aerobically and anaerobically, and hence the main oxidative substrate for mitochondrial TCA.

Evidence that L-LAC transport and metabolism can occur in brain mitochondria was demonstrated for the first time in 2007 when mitochondria from cerebellar granule cells were shown to metabolize externally added L-LAC [14]. This has been confirmed [95] and where the occurrence of LDH in the mitochondria of an astrocytic cell line was shown. In 2008 Hashimoto et al. [66] proposed that neurons contain a mitochondrial lactate oxidation complex that has the potential to facilitate both intracellular and cell–cell lactate shuttles in the brain (see below).

### 5.3. The L-Lactate Mitochondrial Metabolism Plays a Major Role in Cancer Cells

In cancer, L-LAC is the ultimate product of glycolysis (Warburg effect), however, until 2010 its metabolism has not been investigated in detail: in the words of Kennedy and Dewhirst [101] “The implications and consequences of L-LAC utilization by tumors are currently unknown; therefore, future research is needed on the intricacies of tumor metabolism”. No mitochondrial metabolism was proposed until 2010 when de Bari et al. [20] published a paper in which showed that “L-LAC metabolism can occur in normal and cancer prostate cells via the novel mitochondrial L-LAC dehydrogenase”. By using, mainly, the dynamic approach described above, they showed that L-LAC can be transported into mitochondria isolated from both normal (PTNA) and cancer cells (PC3 cells) in a carrier-mediated manner via the putative L-LAC/H^+^ symporter, inhibited by the thiol reagent mersalyl. Inside mitochondria L-LAC is oxidized by the mL-LDH, producing PYR. Normal and cancer cells were found to differ from one another with respect to mL-LDH protein level and activity, being the enzyme more highly expressed and of higher activity in cancer cells. Such a conclusion was confirmed by Hussien and Brooks who found differences in mitochondrial LDH and MCT isoform expression in normal breast cancer and breast cancer cells [102]. Moreover, the kinetic features and pH profiles of the PC3 mL-LDH also differ from those of the PNT1A enzyme, this suggesting the occurrence of separate isoenzymes. Considering the poor oxygen consumption and since fatty acid oxidation is the bioenergetic dominant pathway in the prostate, L-LAC metabolism was suggested to lead to citric cycle anaplerosis to give OAA via pyruvate carboxylase, activated by acetyl-CoA. Citrate could be then formed to be used essentially in fatty acid synthesis in PC3 cells and exported in the extracellular fluid in the PNT1 cells. Two years later, confirmation that L-LAC uptake can trigger metabolic traffic from cytosol to mitochondria and vice versa was found in Hep G2 cells: occurrence of the L-LAC/PYR shuttle (see [9,10]) and the appearance outside mitochondria of OAA, malate and citrate arising from L-LAC uptake and metabolism together with the low oxygen consumption and membrane potential generation were found thus establishing an anaplerotic role for L-Lactate in Hep G2-M for instance in fatty acid synthesis [21]. As emphasized above, the same conclusion was proposed 4 years later for other cancer cells. It was found that Hep G2 cell mitochondria (Hep G2-M) possess an mL-LDH restricted to the inner mitochondrial compartments as shown by immunological analysis, confocal microscopy and by assaying mL-LDH activity in solubilized mitochondria [21]. Cytosolic and mitochondrial L-LDHs were found to differ from one another in their saturation kinetics. The capability of L-LAC to enter mitochondria was shown by measuring the increase in NAD(P)H fluorescence which takes place as a result of L-LAC addition and by monitoring the mitochondrial swelling in ammonium L-LAC solution. Interestingly, in the same experiment, PYR proved to be a non-penetrant metabolite, this suggesting the impossibility that the Cori cycle could occur in these cells. Accordingly, Passarella and Schurr [103] published “L-lactate transport and metabolism in mitochondria of Hep G2 cells-the Cori Cycle revisited”, in which, due to the lack of the PYR carrier activity in cancer mitochondria, it is proposed that gluconeogenesis in Hep G2 cells depends on L-LAC mitochondrial transport, where OAA is formed and exported for gluconeogenesis likely via the L-LAC/OAA antiporter. Recently, it was proposed to include the mitochondrial metabolism of L-LAC in cancer metabolic reprogramming [104].

### 5.4. A Quick, Easy Protocol to Investigate the L-Lactate Transport and Metabolism in Muscle Mitochondria

To ascertain the occurrence of L-LAC transport and metabolism in coupled muscle mitochondria a simple protocol to be used was proposed by Passarella and colleagues who published “The mitochondrial L-lactate dehydrogenase affair” [22] in which the occurrence of an L-LDH and L-LAC metabolism in isolated rabbit gastrocnemius mitochondria was shown. Use was made of the experimental strategy required to show if and how L-LAC can enter mitochondria for subsequent metabolism. Rabbit gastrocnemius muscle was rapidly isolated (5–10 min) following killing the animal and immediately placing it in ice-cold KCl medium (0.1 KCl, 50 mM Tris-HCl, 5 mM MgCl_2_, 1 mM EDTA, 1 mM ATP, pH 7.5). Mitochondria (RGM) were isolated with the exclusion of protease treatment (which proved to result in mitochondrial uncoupling and damage of the mitochondrial carriers, thus making any study of mitochondrial transport impossible) and immediately checked for their intactness by assuring that no reduction in absorbance at 334 nm occurred upon NADH addition. m-L-LDH activity was found in RGM solubilized with 0.1% Triton X-100 (TX-100) as the decrease in absorbance of NADH after PYR addition. No absorbance change should occur when PYR is added to intact mitochondria; clearly indicating that m-L-LDH is in the inner mitochondrial compartment. Surprisingly, such a simple assay was not reported in a paper in which the existence of m-L-LDH was argued against because “the distribution of L-LDH activity, among the fractions paralleled that of PK” [105]. Unfortunately, the occurrence of a mitochondrial PK was later shown by Pizzuto et al. [57,58]. In another study, L-LDH activity was considered to be negligible [106]. Thus, we opine that it is easy to dismiss that m-L-LDH is in the outer mitochondrial membrane/intermembrane space: no NADH oxidation occurs when PYR is added to purified mitochondria, whereas treatment of mitochondria with TX-100 results in NADH oxidation via Complex I with rate increased by the addition of PYR, which reacts with NADH via m-L-LDH.

The existence of an m-L-LDH localized in the inner mitochondrial compartment was simply established by showing the ability of externally added L-LAC to reduce the intramitochondrial NAD^+^. The involvement of L-LDH in this process was confirmed by its inhibition with oxamate, an inhibitor of LDH.

Consistently with the spectroscopic outcome, a static immunological analysis has shown that mitochondria free of cytosolic contamination (no tubulin, a marker of the cytosolic fraction) contain a protein recognized by the L-LDH antibody. As noted above, such a static measurement by itself cannot afford one to determine in which mitochondrial compartment mL-LDH is located.

One wonders why the investigators who have firmly denied the existence of mitochondrial L-LDH did not carry out these simple experiments. Perhaps their view was colored by the mistaken belief, based on incorrect thermodynamic arguments, that mitochondria cannot import L-LAC. Indeed, both Rasmussen et al. [105] and Sahlin et al. [107] argue that the idea of L-LAC conversion to PYR inside mitochondria is not feasible based on thermodynamic principles. They point to a much higher reduction in the NAD^+^/NADH redox couple inside mitochondria; so much higher in fact that it would theoretically eliminate the possibility of L-LAC to PYR conversion. Sahlin et al. [107] went on to suggest that if m-L-LDH was present in the mitochondrial matrix, it would lead to a futile cycle in which PYR would be reduced to L-LAC in mitochondria and vice versa in the cytosol, oxidizing mitochondrial NADH and finally removing the driving force for the electron transport chain. However, given that in brain mitochondria the NAD^+^ concentration is 8–20-fold higher than that of NADH and that PYR is actively oxidized via pyruvate dehydrogenase, it was suggested [14] that m-L-LDH in vivo essentially catalyzes L-LAC oxidation. Ultimately, the removal of the oxidation product by carrier-mediated transport and mitochondrial metabolism overcomes any thermodynamic difficulty. Hence, those results are consistent with the postulate/proposal of Schurr et al. [81,82] that L-LAC is the only major product of cerebral glycolysis and that it can be metabolized inside mitochondria. On the other hand, glucose oxidation to L-LAC is expected to occur when oxidative phosphorylation is reduced since citrate and/or other citric acid cycle intermediates are required outside mitochondria for anabolism to occur (e.g., see [21]). Under such conditioned anaerobic glycolysis is expected to provide ATP and L-LAC in mitochondria to play an anaplerotic role and/or to be transferred to other cells in the intercellular shuttle.

The results of these studies clearly require us to revise the standard, dogmatic view of glucose metabolism via glycolysis and oxidative phosphorylation. Surprisingly, the overwhelming evidence for the location of mL-LDH inside mitochondria has not yet been accepted by the “mL-LDH non-believers”. Additional possible explanations for the failure of the “m-L-LDH non-believers” to monitor L-LAC mitochondrial metabolism in muscle can be proposed. In those studies, skeletal muscle mitochondria were investigated. It is our opinion that the investigators involved were unable to isolate coupled mitochondria, a task, which is not easy, where skeletal muscle samples are concerned, and that they may not be careful enough in selecting reaction media and the use of inhibitors at the right concentrations. For instance, the failure to measure oxygen consumption as a result of L-LAC addition to skeletal muscle mitochondria [108] could be due to the presence of 5 mM MgSO_4_ in the medium used to prepare isolated skeletal muscle mitochondria, where sulfate is known to enter mitochondria and to cause efflux of intramitochondrial phosphate, malate, and succinate [109].

Moreover, 60 mM lactobionate included in the medium used to measure oxygen uptake by mitochondria is expected to prevent L-LAC uptake due to its chemical structure and high concentration. Finally, no control samples were reported of c-L-LDH and m-L-LDH sensitivity or insensitivity to 60 mM lactobionate. Notice that 60 mM lactobionate was used in a paper in which lactate oxidation was proposed to occur outside mitochondria [110]. Of course, it is impossible to mimic cytosol totally with the medium used for in vitro experiments, however, control must be made that the absence of L-LAC metabolism is not dependent on the composition of the media used in the experiments as well as on the mitochondria isolation.

### 5.5. Achievements in the L-Lactate-Mitochondria Affair

Although the role of L-LAC in energy metabolism has been revisited at the beginning of this century, whether and how mitochondria can take up and metabolize L-LAC is still ignored in the literature concerning both normal and cancer cells. Surprisingly enough, in many papers dealing with mitochondria and cancer published in the last decade [4,8,111,112,113,114,115,116,117,118,119,120,121,122,123,124,125,126,127,128,129,130,131,132] no mention of the L-LAC metabolism in mitochondria was made this confirming that incorrect use of the Web database can result in incorrect information for the readers [133] and raising serious doubts about the reliability of incomplete information.

On the other hand, recently, Fulghum et al. [134] concluded that mLDH does not “contribute to cardiac bioenergetics in mice”. No mention of the papers of the authors of this review was made. Contrarily, in 2020 Young et al. report [135] “Using the guide supplied by Passarella et al., [22] we counter the conclusions drawn by Fulghum et al. and demonstrate that mitochondria oxidize lactate”. It was a great surprise that in 2020 Borst reported “I concur with the sceptics and disregard matrix mitoLDH until proven wrong by rigorous experiments” [47]. Indeed “*Quandoque bonus dormitat Homerus*”! (even the great Homer sometimes dozes). An even greater surprise was that the paper containing this sentence has been published without any citation of the work of the authors of this review.

Therefore, in this review we will try to provide definitive answers to several specific questions, to offer closure to the L-LAC-mitochondria affair.

- Question 1: Do L-LAC transport and metabolism occur in mitochondria? The answer here is yes, they do. The existence of an mL-LDH was definitively confirmed in mammalian, plant, and yeast mitochondria (see [103]) with its existence being finally recognized by the inclusion of mL-LDH in the MitoCarta: (http://www.broadinstitute.org/pubs/MitoCarta/index.htrnl) (accessed 20 November 2021).

- Question 2: Do L-LAC and PYR enter mitochondria via the same carrier? The answer here is no since there are separate carriers!

The mechanism by which L-LAC enters mitochondria has often not been considered or it is assumed that L-LAC enters mitochondria via the PYR transporter. However, in RLM it was shown that two separate carriers transport PYR and L-LAC into mitochondria. Although α-cyano-hydroxycinnamate can inhibit the uptake of both PYR and L-LAC, the PYR carrier is inhibited at a concentration (25 µM) at which no inhibition of L-LAC transport occurs. Moreover, other antiports for L-LAC have been shown to occur: comparison made among the inhibition profiles of L-LAC transport/transporters due to non-penetrant compounds shows that the L-LAC carriers, (the L-LAC/H^+^ symporter, the L-LAC/PYR, and L-LAC/OAA antiporters), the PYR carrier and other carriers, including those that transport D-LAC, all differ from one another [19].

- Question 3: In which of the mitochondrial compartments is L-LDH located?

The mitochondrial L-lactate is in the mitochondrial matrix. That L-LAC enters mitochondrial matrix where is oxidized by the mitochondrial L-LDH has been shown using many experimental approaches:

(1) Swelling measurements exhibiting the stereospecificity of the process and the inhibition of swelling found due to non-penetrant. Obviously, a carrier-mediated transport itself postulates that L-LAC is metabolized inside mitochondria.

(2) Measurements of an increase in the redox state of the intramitochondrial pyridine nucleotides found because of the addition of L-LAC to mitochondrial samples in the absence of external NAD^+^ indicate that mitochondrial metabolism occurs inside the organelles via the NAD^+^-dependent mL-LDH.

(3) Measurements of oxygen consumption by coupled purified mitochondria upon L-LAC addition inhibited by both rotenone, inhibitor of complex I, and oxalate/oxamate, inhibitors of L-LDH.

(4) Proton efflux and the increase in membrane potential were found because of L-LAC uptake and metabolism. Conversely, proton uptake occurs because of L-LAC addition to mitochondria previously treated with an inhibitor cocktail used to prevent any energy metabolism, due to the L-LAC/H^+^ symport.

(5) Measurements show the efflux of a variety of metabolites newly synthesized inside mitochondria due to externally added L-LAC.

Last, but not least, (a) enzymatic assays (b) immunological analysis, and (c) confocal fluorescence microscopy can be used: (a) allows for initial information about enzyme kinetics features; (b) and (c) can reveal the existence of m-L-LDH even if inactive but have the limitation that they are of no value in dissecting L-LAC metabolism. The fluorescence microscopic studies by Lemire et al. [136] further revealed the localization of LDH within the mitochondria. In that study, it was reported that “mitochondrial fractionation and western blot analysis revealed that LDH1 isozyme populated predominantly the matrix/inner membrane components. “Thus, in distinction with [79], we claim that the localization of the mitochondrial L-LDH is no longer a matter of debate.

Question 4: Can cancer energy metabolism be described exhaustively with no consideration for L-LAC transport and metabolism in mitochondria? The answer here is no (and see above).

We hope that all the sceptics can accept the above conclusion as any good student of ours did!

Interestingly, in distinction with that reported by Brooks et al. [137] after 2014 when “The mitochondrial L-lactate dehydrogenase affair” was published [22], to the best of our knowledge, with the exception of Fulghum et al. [134], there was no denier of the existence of the mitochondrial L-LDH.

## Figures and Tables

**Table 1:** Static approach to investigate the role of mitochondrial transport in energy metabolism. Pro vs. Contra.

Biological Sample	Approach	Pro vs. Contra
Tissue/cell	Carrier protein/enzyme purification	Pro: can give evidence for the existence and study of metabolite binding to the carrier/enzyme Contra: NATM
Proteins, lipids	Reconstitution study	Pro: can show the capability of the isolated protein to work as a carrier when present in proteoliposomes/of the isolated enzyme to catalyze the reaction Contra: NATM
Proteins	Sequencing	Pro: can give the possibility to investigate carrier protein/enzyme structure and metabolite binding sites Contra: NATM
Cell/cell component	Cell fractionation	Pro: can show both carrier protein/enzyme localization and identification of the organelles involved in transport/catalysis Contra: NATM
No biological system	Bioinformatic analysis	Pro: can give a first predictive insight into the localization and the role of a protein and in comparative genetics Contra: NATM
Cell/cell component	Immunological analysis	Pro: can give evidence for the existence and the amount of a protein independently on its catalytic properties and localization Contra: NATM
Cell/cell component	Confocal microscopy	Pro: can show localization of a protein in vitro Contra: NATM
Cell/cell component	Magnetic resonance spectroscopy	Pro: allows to identify the levels of some metabolites in specific anatomical structures, Contra: NATM
Cell/cell component	High-resolution mass spectroscopy	Pro: can show that enriched substrate can enter mitochondria by using ^13^C and 2-^2^H labels Contra: NATM
Cell/cell component	Transmission electron microscopy	Pro: can show the mitochondrial localization Contra: NATM
Cell/cell component	High-performance liquid chromatography (HPLC)	Pro: can give qualitative/quantitative analysis in biological sample Contra: NATM

Abbreviation: NATM, nothing about transport and metabolism both in vitro and in vivo.

**Table 2:** The compound/metabolite detection outside mitochondria.

Detected Compound/Metabolite	Detecting Systems	A_340_ Changes
PEP	NADH, ADP, pyruvate kinase, L-lactate dehydrogenase	Decrease
OXALOACETATE	NADH, malate dehydrogenase	Decrease
PYRUVATE	NADH, L-lactate dehydrogenase	Decrease
ASPARTATE	NADH, 2-oxoglutarate, aspartate aminotransferase, malate dehydrogenase	Decrease
GLUTAMATE	NAD^+^, glutamate dehydrogenase	Decrease
MALATE	NADP^+^, malic enzyme	Decrease
CITRATE	NADH, coenzyme A, ATP, malate dehydrogenase, citrate lyase	Decrease
ADP	NADH, PEP, pyruvate kinase, L-lactate dehydrogenase	Decrease
ATP	NADP^+^, glucose, hexokinase, glucose-6-phosphate dehydrogenase	Decrease

## Data Availability

Not applicable.
